# Comparative effectiveness of acupuncture and Tuina for cervical vertigo: a systematic review and network meta-analysis of randomized controlled trials

**DOI:** 10.3389/fneur.2026.1691312

**Published:** 2026-05-08

**Authors:** Zhijun Yang, Penghui Li, Lin Jiang, Yu Xu, Yuzhe Zhang, Lixiang Yuan, Jie Li, Chuanshi Lei, Kangyan Zhai, Youkang Dong

**Affiliations:** 1 Second Clinical Medical College, Yunnan University of Chinese Medicine, Kunming, Yunnan, China; 2 First Clinical Medical College, Yunnan University of Chinese Medicine, Kunming, Yunnan, China; 3 College of Ethnic Medicine, Yunnan University of Chinese Medicine, Kunming, Yunnan, China; 4 Department of Tuina, The First Affiliated Hospital of Yunnan University of Chinese Medicine, Kunming, Yunnan, China; 5 Department of Acupuncture, Zhenkang County Hospital of Traditional Chinese Medicine, Lincang, Yunnan, China; 6 Department of Rehabilitation, Lincang Hospital of Chinese Medicine, Lincang, Yunnan, China

**Keywords:** acupuncture and tuina, cervical vertigo, efficacy, network meta-analysis, systematic review

## Abstract

**Objective:**

This study conducted a network meta-analysis to systematically evaluate and compare the effectiveness of acupoint-based stimulation and manual therapies for cervicogenic vertigo. We examined needling-based stimulation, including manual acupuncture and electroacupuncture; thermal stimulation through moxibustion, including warm-needle techniques; acupotomy; manual therapy including tuina and Chinese osteopathic manipulation; and prespecified combinations of these approaches. By synthesizing evidence from parallel-group randomized controlled trials, we aimed to provide an evidence-based foundation for clinical decision-making and practice.

**Methods:**

We systematically searched China National Knowledge Infrastructure, Wanfang Data, VIP Chinese Science and Technology Journal Database, PubMed, Embase, the Cochrane Library, Web of Science, and Ovid MEDLINE to identify eligible parallel-group randomized controlled trials. We performed a systematic review and network meta-analysis to evaluate the efficacy and safety of acupoint-based stimulation and manual therapy strategies, including manual acupuncture and electroacupuncture, moxibustion, acupotomy, Tuina and Chinese osteopathic manipulation, and prespecified combinations. We assessed outcomes using the Evaluation Scale for Cervical Vertigo, the Dizziness Handicap Inventory, and overall response measured as the total effectiveness rate. This study was registered in the International Prospective Register of Systematic Reviews (PROSPERO; CRD420251113507).

**Results:**

Sixty-six parallel-group randomized controlled trials involving 5,797 patients and 18 intervention combinations were included. A combination of electroacupuncture and Tuina showed the most significant overall clinical benefit compared with Tuina alone (OR = 1.53; 95% CI: 1.23–1.89). The following highest effects were observed for a combination of electroacupuncture and moxibustion (OR = 1.52; 95% CI: 1.23–1.87) and a combination of electroacupuncture and acupotomy (OR = 1.50; 95% CI: 1.17–1.92). Electroacupuncture alone also outperformed Tuina (OR = 1.36; 95% CI: 1.12–1.67). For the Evaluation Scale for Cervical Vertigo, a combination of manual acupuncture and Chinese osteopathic manipulation produced the most significant improvement compared with tuina (mean difference = 12.52; 95% confidence interval: 8.51–16.54). A combination of manual acupuncture and moxibustion also significantly improved the Evaluation Scale for Cervical Vertigo vs. tuina (mean difference = 12.34; 95% confidence interval: 8.77–15.90), although the effect was slightly smaller. Among monotherapies, manual needle acupuncture was superior to tuina in improving the Evaluation Scale for Cervical Vertigo (mean difference = 8.38; 95% confidence interval: 4.66–12.11). For the Dizziness Handicap Inventory, warm needle moxibustion showed the largest reduction in dizziness-related disability compared with tuina (standardized mean difference = 2.04; 95% confidence interval: 1.49–2.58).

**Conclusions:**

Acupuncture- and Tuina -based interventions may improve outcomes in cervicogenic vertigo; however, comparative rankings should be interpreted cautiously, given the predominance of low/very low certainty evidence. Combination regimens, particularly electroacupuncture plus tuina, tended to rank favorably for overall response, while manual acupuncture combined with Chinese osteopathic manipulation appeared promising for the Evaluation Scale for Cervical Vertigo; evidence for the Dizziness Handicap Inventory remains sparse. High-quality, internationally conducted head-to-head randomized controlled trials using validated outcomes and rigorous bias control are needed to confirm comparative effectiveness.

**Systematic review registration:**

https://www.crd.york.ac.uk/prospero/display_record.php?ID=CRD420251113507, identifier: CRD420251113507.

## 1 Introduction

Cervical Vertigo (CV) is a dizziness syndrome caused by structural or functional abnormalities of the cervical spine and surrounding tissues (1, 2).

The clinical presentation is characterized by episodes of vertigo lasting minutes to hours, which may become chronic in some patients. While the sensation is most frequently described as “non-rotational” dizziness, it can also manifest as rotational vertigo. These symptoms are commonly triggered or worsened by head movements, particularly rotation or extension (3).

In addition to dizziness, patients frequently present with neck and shoulder pain, headache, nausea, tinnitus, numbness or paresthesia in the upper limbs, and even psychological symptoms such as anxiety and depression. In rare but severe cases, sudden falls may occur (4). Episodes of cervical vertigo are frequently accompanied by prominent autonomic symptoms, including nausea, vomiting, urgency of urination or defecation, changes in skin color, and hyperhidrosis. These may be combined with cochlear symptoms such as tinnitus, aural fullness, and hearing loss, as well as visual disturbances like blurred vision, floaters, and photophobia (5).

With changes in modern lifestyle, including accelerated daily rhythms and increasing psychological and physical stress, the incidence of CV has been rising markedly. The concept of cervical vertigo was first proposed by Barré in 1926 and later expanded upon by Ryan and Cope (6). Epidemiological data suggest that the prevalence of cervical spondylosis in China ranges from 3.8 to 17.6%, and approximately 50% of these patients experience varying degrees of vertigo (7). Due to the close relationship between cervical vertigo and cervical spine function, clinical presentations can be highly variable, posing significant diagnostic challenges (8).

In clinical settings, CV affects a large population and substantially impairs patients' quality of life and occupational functioning. Diagnosis primarily relies on patient-reported symptoms and requires collaboration among neurology, otolaryngology, and Traditional Chinese Medicine (TCM) departments, particularly acupuncture and Tuina practitioners. Prolonged forward-leaning posture and sedentary habits associated with modern work environments can lead to sustained cervical traction, accelerating degenerative changes and dysfunction, thereby increasing the risk of CV (9).

Symptom severity varies by individual, ranging from mild dizziness to incapacitating vertigo that significantly disrupts daily life and productivity. The pathological mechanism of cervical vertigo in modern medicine is complex. The prevailing view attributes the condition principally to impaired central sensory integration resulting from disordered cervical proprioceptive input, while also acknowledging the potential involvement of factors such as vertebral artery hemodynamic alterations, heightened sympathetic nerve activity, and cervicoscapular myofascial pain syndrome. This complexity contributes to the therapeutic challenge (10).

Traditional Chinese Medicine posits that the root cause of vertigo lies in wind, phlegm, blood stasis, or deficiency agitating the clear orifices of the head. The principle of treatment, therefore, is to employ external methods that stimulate designated acupoints and meridians. This serves to harmonize qi-blood circulation, resolve stasis and obstruction, and ultimately recover the body's innate balance. Current treatments fall into two broad categories: Western medicine and Traditional Chinese Medicine. Western medical approaches include pharmacological therapies, such as calcium channel blockers, vasodilators, and vestibular sedatives, as well as surgical interventions like percutaneous laser disc decompression and anterior cervical fusion (11). However, these methods often carry a high risk of recurrence, poor patient adherence, and a relatively high rate of adverse effects.

In contrast, TCM provides both internal therapies, such as herbal decoctions, and external modalities. For CV, commonly used external treatments include various forms of acupuncture like needle acupuncture, electroacupuncture, and warm needling, as well as moxibustion and Tuina, which is Chinese manual therapy. While internal therapies are recognized for their therapeutic efficacy, they often exhibit a delayed onset of action. External treatments, on the other hand, are highly regarded for their simplicity, rapid therapeutic effect, cost-efficiency, and strong patient compliance. Among these, acupuncture and Tuina are the most extensively utilized external interventions for cervical vertigo, owing to their favorable safety profile, hygienic administration, and operational convenience. For instance, needle acupuncture and moxibustion are applied to specific acupoints to regulate Qi and blood flow, dispel pathogens, and restore balance. Tuina aims to relax muscles, free joint movement, and clear meridian obstructions. These modalities can be used alone or in combination. However, clinical responses to these therapies can vary markedly across patients, primarily due to heterogeneous clinical practices and the absence of unified, evidence-based treatment guidelines.

Therefore, the optimization and comparative evaluation of diverse acupuncture and tuina strategies represent a critical area of ongoing research. This study undertakes a Stata network meta-analysis of existing randomized controlled trials (RCTs) to enable indirect comparisons of the efficacy and safety of various acupuncture and tuina protocols in the management of cervical vertigo. By establishing a hierarchical ranking of treatment effectiveness, the study aims to generate robust, evidence-based recommendations to inform clinical decision-making and enhance the standardization of Traditional Chinese Medicine interventions for cervical vertigo.

## 2 Materials and methods

This network meta-analysis (NMA) was performed in accordance with the Preferred Reporting Items for Systematic Reviews and Meta-Analyses extension statement for network meta-analyses (PRISMA-NMA; refer to Supplementary Table 1) (12). In the absence of direct comparative evidence from RCTs on different acupuncture and Tuina therapies, a network meta-analysis was applied to combine direct and indirect evidence from RCTs, enabling probabilistic predictions of their efficacy ranking (13).

To ensure methodological transparency, scientific rigor, and reproducibility, the study protocol was prospectively registered in the International Prospective Register of Systematic Reviews (PROSPERO; CRD420251113507).

### 2.1 Data sources and search strategy

A comprehensive literature search was systematically conducted across the following electronic databases: China National Knowledge Infrastructure (CNKI), Wanfang Data, VIP Chinese Science and Technology Journal Database (VIP), PubMed, Embase, the Cochrane Library, Web of Science, and Ovid MEDLINE. The search encompassed studies published from the inception of each database up to July 1, 2025. Free-text terms and controlled vocabulary (e.g., MeSH terms) were combined to optimize search sensitivity and precision, with no language restrictions applied. The following keywords and their synonyms were used:

“cervicogenic vertigo,” “cervical dizziness,” “cervical vertigo,” “cervical spondylopathy of the vertebroarterial type,” “acupuncture,” “acupuncture treatment,” “acupuncture therapy,” “pharmacoacupuncture therapy,” “pharmacoacupuncture treatment,” “acupotomy,” “needle knife,” “needle scalpel,” “moxibustion,” “electroacupuncture,” and “massage.”

Search strategies were customized for each database to maximize both sensitivity and specificity in the identification of eligible studies.

### 2.2 Selection criteria

#### 2.2.1 Inclusion criteria

Studies were included in this review if they satisfied the following conditions:

##### 2.2.1.1 Diagnostic criteria

Eligible studies must have diagnosed cervical vertigo based on at least one of the following standards:

- Multidisciplinary expert consensus on the diagnosis and treatment of vertigo (14);
- Summary of the second national symposium on cervical spondylosis (15);
- Summary of the third national symposium on cervical spondylosis (16);
- Expert consensus on the diagnosis and treatment of vertigo (17);
- Criteria for the diagnosis and therapeutic effect of TCM syndromes and diseases (18).

Study design: We included only parallel-group randomized controlled trials; throughout the manuscript, we refer to these studies as RCTs.

Interventions: We included studies of acupuncture- and Tuina-related regimens covering two categories of interventions, acupoint-based stimulation and manual therapy. Eligible interventions comprised acupuncture, including manual acupuncture and electroacupuncture; moxibustion-based therapies, including warm-needle techniques; manual therapy, including Tuina and Chinese osteopathic manipulation; acupotomy; medication; and prespecified combinations.

Chinese osteopathic manipulation is a joint-directed manual therapy intended to correct minor cervical facet dysfunction. Clinicians typically perform palpation-based assessment and then apply joint-specific adjustment or mobilization techniques, such as traction, rotation, and flexion–extension maneuvers, with or without adjunct soft-tissue work (19). We coded an intervention as Chinese osteopathic manipulation when trials explicitly used terms such as bone-setting, reduction, or repositioning and described joint-directed adjustment procedures; we coded protocols described only as general Tuina or massage without joint-directed adjustment or reduction as Tuina.

For studies derived from the same patient cohort, only the most complete or most recently published version was retained.

##### 2.2.1.2 Outcome measures

Eligible RCTs must have reported at least one of the following outcomes:

Evaluation Scale for Cervical Vertigo (ESCV): Mean differences in scores before and after treatment, reflecting vertigo severity and cervical function, with corresponding standard deviations.

Dizziness handicap inventory (DHI): Mean differences in DHI scores, measuring dizziness severity, postural imbalance, impact on daily activities, and fall risk, including standard deviations.

Total effectiveness rate: A post-treatment composite response derived from categorical efficacy ratings (e.g., cured/markedly effective/effective/ineffective), calculated as the proportion of participants classified as “effective” (all categories other than “ineffective,” according to each trial's prespecified threshold) among all participants. Given heterogeneity in response-threshold definitions across trials, we grouped operational definitions into nine categories (cov1 = 1–9; Table S1 in Data Sheet 1) and examined their influence using network meta-regression.

#### 2.2.2 Exclusion criteria

Studies were excluded based on the following conditions:

- Non-randomized studies, including meta-analyses, narrative reviews, systematic reviews, data mining reports, conference abstracts, and other publications not based on RCTs.
- RCTs with ambiguous outcome measures or insufficient quantitative data required for meta-analysis.
- Gray literature or studies deemed to be of low methodological quality.
- To improve clinical comparability and satisfy the transitivity assumption in network meta-analysis, we restricted acupuncture to invasive body-needle stimulation and excluded scalp acupuncture, auricular acupuncture, transcutaneous acupoint stimulation, and laser acupuncture.
- We excluded these modalities because they differ substantially from body-needle acupuncture in the target stimulation site of interest, namely the cervical region, in the pathway through which effects may arise, and in stimulus dosimetry; including them could increase heterogeneity and weaken network connectivity.

Prior to final inclusion, all studies underwent an initial screening of titles and abstracts. Full-text articles of potentially eligible RCTs were independently assessed by two reviewers. Any discrepancies were resolved through discussion or, when necessary, consultation with a third reviewer. To maintain data integrity, only the most recent and comprehensive version was retained in cases of overlapping or duplicate data sources.

### 2.3 Data extraction and quality assessment

Data extraction was independently conducted by three reviewers following the Preferred Reporting Items for Systematic Reviews and Meta-Analyses (PRISMA) guidelines. Any disagreements were resolved through group discussion, with the involvement of a fourth author to reach consensus.

From each eligible randomized controlled trial, the following data were extracted: first author, year of publication, sample size, participants' age, disease duration, intervention details, and reported outcome measures.

The methodological quality of the included RCTS was evaluated using the Cochrane Risk of Bias Tool (Version 2.0) (Cochrane Collaboration, London, UK) (20). This tool assesses potential sources of bias across five domains:

- Bias arising from the randomization process;
- Bias due to deviations from intended interventions;
- Bias due to missing outcome data;
- Bias in the measurement of outcomes;
- Bias in the selection of the reported result.

Each domain was independently assessed and classified as having either a low risk of bias, high risk of bias, or some concerns, according to the criteria outlined in the *Cochrane Handbook for Systematic Reviews of Interventions*.

#### 2.3.1 Outcome measures and statistical indicators

The primary outcome was the total effectiveness rate, while secondary outcomes included scores from the Evaluation Scale for Cervical Vertigo (ESCV) and the Dizziness Handicap Inventory (DHI).

#### 2.3.2 Software and data management

All references were managed using EndNote X20 (Clarivate Analytics, Philadelphia, PA, USA), and data extraction was carried out using Microsoft Excel 2024 (Microsoft Corporation, Redmond, WA, USA). The network meta-analysis was performed using Stata version 17.0 (StataCorp LLC, College Station, TX, USA).

#### 2.3.3 Effect size metrics

For binary outcomes, we used odds ratios (ORs) with 95% confidence intervals (CIs). For continuous outcomes, we used mean differences (MDs) with 95% CIs; standardized mean differences (SMDs) were used only when the same outcome was measured using different scales.

In the network evidence diagram, the size of each node represents the sample size of the corresponding intervention, while the thickness of the connecting lines reflects the number of RCTs comparing each pair of interventions.

When the network structure forms open loops, a consistency model is applied by default. For closed-loop structures, inconsistency tests are conducted to evaluate the agreement between direct and indirect evidence for each outcome measure. If *P* > 0.05, it indicates good consistency, and the consistency model is retained. If *P* ≤ 0.05, inconsistency is suspected, and subgroup analyses or meta-regression are performed to explore sources of heterogeneity.

#### 2.3.4 Ranking and bias assessment

To summarize treatment rankings across all interventions, we calculated the surface under the cumulative ranking curve (SUCRA) values and displayed them using cumulative ranking probability plots.

For closed loops in the network, Loop-specific tests showed that the inconsistency factors were close to zero and their 95% confidence intervals included zero, indicating no important local inconsistency. A CI that includes zero indicates good agreement between direct and indirect estimates.

Finally, comparison-adjusted funnel plots were constructed to evaluate potential publication bias and small-study effects.

### 2.4 Certainty of evidence (GRADE and CINeMA)

We assessed certainty of evidence using the Grading of Recommendations Assessment, Development and Evaluation (GRADE) approach and the Confidence in Network Meta-Analysis (CINeMA) framework. Because all included studies were RCTs, we started at high certainty and downgraded across six domains: within-study bias, indirectness (transitivity), imprecision, heterogeneity, incoherence, and across-study bias (publication bias/small-study effects). We assessed within-study bias with Cochrane risk of bias tool for randomized trials (RoB 2) and the CINeMA contribution matrix; evaluated imprecision using 95% confidence intervals and, where applicable, minimal important differences; quantified heterogeneity using τ^2^ and prediction intervals when available; examined incoherence with node-splitting in closed loops and clinical/methodological judgment otherwise; and assessed across-study bias using registration status, gray-literature searches, and comparison-adjusted funnel plots. We rated each domain as no concerns, some concerns, or major concerns and downgraded by 0–2 levels to assign an overall certainty of high, moderate, low, or very low. Results are presented in Tables S3 and S4 in Data Sheet 1.

## 3 Results

### 3.1 Systematic review and characteristics of the included studies

**Figure 1:**
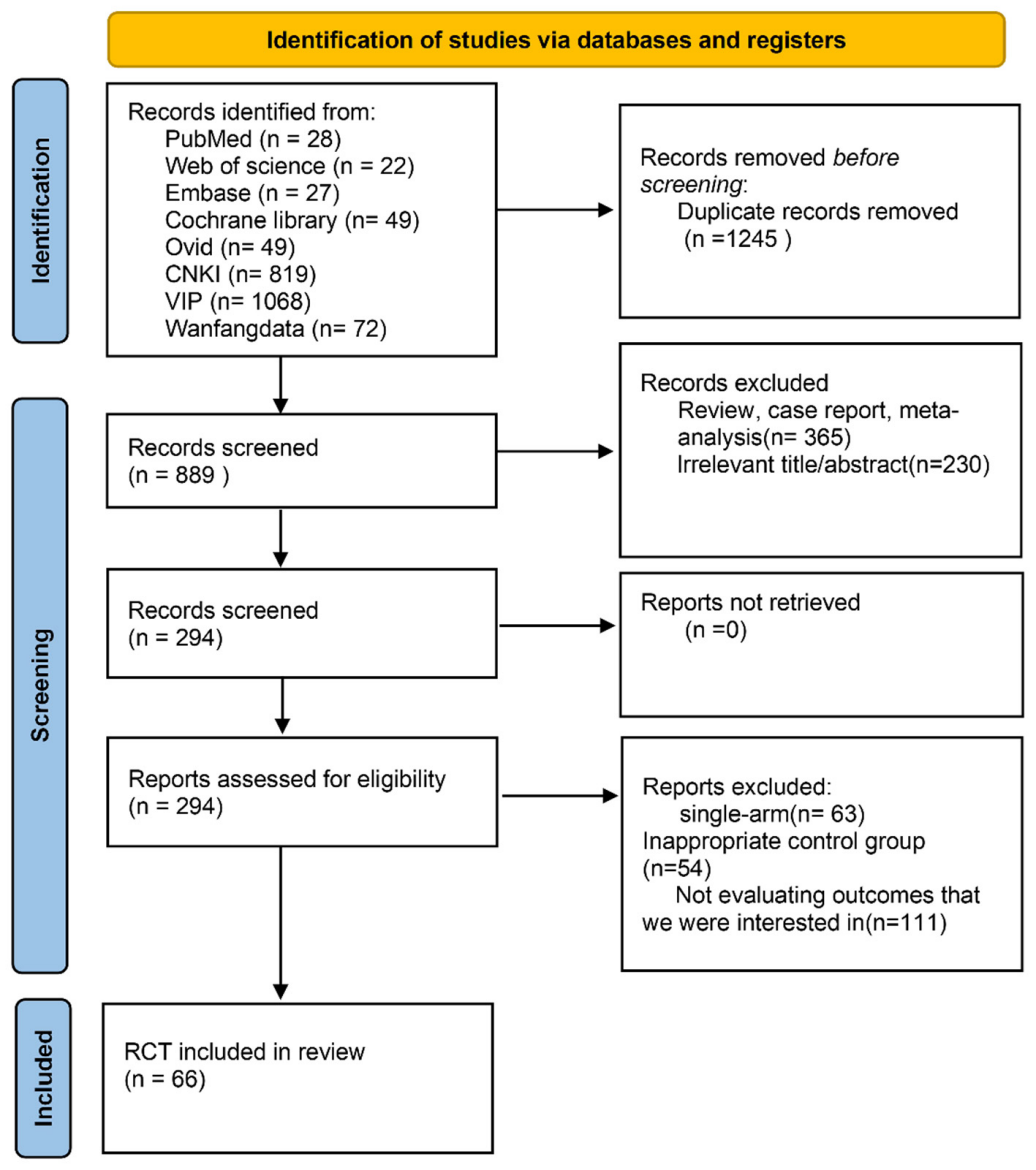
Flow diagram of the study selection process based on the PRISMA (preferred reporting items for systematic reviews and meta-analyses) guidelines.

A total of 2,134 records were initially identified through electronic database searches. After the removal of duplicates and the screening of titles and abstracts, 889 articles were retained for full-text assessment. Following a rigorous evaluation against the predefined inclusion and exclusion criteria, a final set of eligible studies was included in the meta-analysis (Figure 1).

A total of 5,797 patients were enrolled across the included studies and received one of the following 18 intervention strategies: manual needle acupuncture (AP); a combination of acupuncture and acupotomy (AP–APT); a combination of acupuncture and Chinese osteopathic manipulation (AP–COM); a combination of acupuncture and medication (AP–MED); a combination of acupuncture and moxibustion (AP–MOX); a combination of acupuncture and Tuina (AP–TUI); a combination of acupuncture, Tuina, and medication (AP–TUI–MED); Chinese osteopathic manipulation (COM); electroacupuncture (EA); a combination of electroacupuncture and acupotomy (EA–APT); a combination of electroacupuncture and medication (EA–MED); a combination of electroacupuncture and moxibustion (EA–MOX); a combination of electroacupuncture and Tuina (EA–TUI); medication (MED); Tuina (TUI); warm needle moxibustion (WNM); a combination of warm needle moxibustion and acupotomy (WNM–APT); and a combination of warm needle moxibustion and Tuina (WNM–TUI). Detailed characteristics of all included studies are summarized in Tables 1 and 2.

**Table 1:** Baseline characteristics of studies included in the network meta-analysis.

Included in the study	Number of cases/cases		Age (x-±s)		Disease course (x-±s)	
	Experimental Group	Control group	Experimental Group	Control group	Experimental Group	Control group
Cao et al. (28)	48	48	45.7 ± 3.1	46.9 ± 2.9	2.72 ± 1.14 (Y)	2.81 ± 1.23 (Y)
Zeng et al. (29)	48	48	51.21 ± 10.83	50.36 ± 11.16	3.59 ± 1.03 (Y)	3.66 ± 1.15 (Y)
Han (30)	47	47	46.41 ± 5.22	46.37 ± 5.19	5.01 ± 2.04 (Y)	4.96 ± 2.02 (Y)
Hu and Tang (31)	30	30	37.70 ± 7.48	39.23 ± 6.64	15.53 ± 5.56 (Y)	13.13 ± 5.02 (Y)
Yin (32)	14	14	38.02 ± 4.21	37.84 ± 4.19	4.11 ± 1.03 (Y)	4.01 ± 0.97 (Y)
She Ruitao and Yuanfang (33)	31	31	41.5 ± 9.8	39.2 ± 2	5.0 ± 2.9 (Y)	4.8 ± 3.0 (Y)
Miao et al. (34)	48	48	46.65 ± 8.81	47.12 ± 9.30	24.79 ± 6.53 (M)	25.64 ± 7.87 (M)
Ma et al. (35)	46	46	52.58 ± 3.46	52.56 ± 3.45	5.37 ± 1.25 (W)	5.35 ± 1.23 (W)
Li (36)	30	30	40.13 ± 7.06	39.67 ± 6.34	5.53 ± 1.71 (M)	5.70 ± 2.60 (M)
Chen et al. (37)	44	45	47 ± 10	49 ± 13	18.02 ± 4.84 (M)	16.31 ± 4.44 (M)
Hu et al. (38)	39	39	32.38 ± 5.48	33.22 ± 5.17	5.40 ± 0.78 (M)	5.62 ± 0.93 (M)
Yue et al. (39)	32	28	44.61 ± 10.49	37.54 ± 11.61	6.74 ± 4.53 (D)	8.12 ± 6.21 (D)
Cai et al. (40)	57	57	45.60 ± 8.36	46.10 ± 8.12	23.88 ± 7.87 (M)	27.12 ± 8.14 (M)
Wang et al. (41)	148	148	41.01 ± 3.91	41.30 ± 4.58	2.11 ± 0.33 (M)	2.07 ± 0.37 (M)
Chen et al. (42)	30	30	N/A	N/A	N/A	N/A
Wan (43)	30	30	35.7 ± 5.1	35.4 ± 4.9	13.2 ± 7.0 (Y)	11.9 ± 7.5 (Y)
Jiang et al. (44)	35	35	43 ± 14	42 ± 13	3.3 ± 1.7 (M)	3.0 ± 1.3 (M)
Chen et al. (45)	40	40	45.41 ± 5.04	44.21 ± 5.61	134 ± 5.12 (D)	136.17 ± 4.12 (D)
Su (46)	40	40	N/A	N/A	N/A	N/A
Lin et al. (47)	50	50	47.37 ± 10.12	48.21 ± 9.63	23.56 ± 7.04 (M)	22.94 ± 6.37 (M)
Zhuang et al. (48)	21	19	N/A	N/A	N/A	N/A
Zhu Fuping and Yang Shunyi (49)	17	17	53.7 ± 11.9	53.3 ± 11.7	2.90 ± 1.12 (Y)	2.78 ± 1.09 (Y)
Li Yuemei and Li Yanhui (50)	48	40	46	47	382 (D)	379 (D)
Lixia (51)	30	30	47.73 ± 6.62	48.77 ± 7.87	19.40 ± 10.69 (M)	17.00 ± 9.88 (M)
Wei (52)	45	43	35 ± 6.8	32 ± 7.2	N/A	N/A
Jian (53)	47	47	34 ± 5.2	36 ± 6.7	N/A	N/A
Yi et al. (54)	30	30	45.1 ± 5.7	46.1 ± 4.8	5.42 ± 2.15 (Y)	5.27 ± 2.23 (Y)
Huang and Wang (55)	30	30	N/A	N/A	N/A	N/A
Shen et al. (56)	78	60	49.63 ± 8.39	48.36 ± 8.42	21.68 ± 7.33 (M)	22.03 ± 7.65 (M)
Qin and Gu (57)	40	38	52 ± 2	51 ± 2	6.33 ± 0.30 (Y)	6.45 ± 0.40 (Y)
Gong and Wang (58)	30	30	40.88 ± 11.12	42.13 ± 9.48	2.38 ± 1.29 (Y)	1.97 ± 1.11 (Y)
Qiu (59)	50	49	35.90 ± 5.74	37.52 ± 7.16	9.35 ± 3.10 (Y)	9.54 ± 3.17 (Y)
Zhan (60)	65	59	41.64 ± 7.16	40.82 ± 7.42	N/A	N/A
Zhang and Zhang (61)	45	45	53.3	51.8	5.5 (M)	5.8 (M)
Yi (62)	75	75	25.4 ± 1.6	25.9 ± 1.7	4.2 ± 1.1 (M)	4.0 ± 1.2 (M)
Li (63)	31	23	41.86	44.26	2.37 (Y)	2.87 (Y)
Li Zhiqin (64)	41	39	48.1 ± 20.4	47.8 ± 16.4	6.3 ± 2.1 (M)	6.4 ± 2.1 (M)
Fan Dehui (65)	65	59	41.64 ± 7.16	40.82 ± 7.42	N/A	N/A
Min and Zhang (66)	56	55	50.6	48.2	N/A	N/A
Chen (67)	47	47	49.15 ± 4.22	48.97 ± 4.16	N/A	N/A
Teng and Duan (68)	69	70	48.2 ± 14.6	46.4 ± 15.3	N/A	N/A
Guo and He (69)	60	60	38.6	36.3	N/A	N/A
Yu (70)	34	34	N/A	N/A	N/A	N/A
Wu et al. (71)	35	35	54.3	53.5	5.1 (Y)	4.8 (Y)
Guo and Yan (72)	50	50	53.68 ± 5.14	52.42 ± 8.96	21.9 ± 13.91 (M)	19.24 ± 13.44 (M)
Luo et al. (73)	47	46	55 ± 4	58 ± 4	12.0 ± 2.6 (M)	10.1 ± 2.5 (M)
Yu et al. (74)	33	32	46.3 ± 4.5	47.1 ± 5.5	2.6 ± 1.3 (Y)	4.5 ± 1.7 (Y)
Deng (75)	64	50	51.5 ± 6.1	48.1 ± 6.5	38.2 ± 5.6 (D)	36.2 ± 7.1 (D)
Lin et al. (76)	50	50	56.64 ± 9.87	57.16 ± 9.42	2.43 ± 2.82 (Y)	2.46 ± 2.39 (Y)
Hua and Li (77)	60	60	N/A	N/A	N/A	N/A
Wu et al. (78)	44	42	58 ± 13	62 ± 13	3.81 ± 0.59 (Y)	3.54 ± 0.55 (Y)
Wang et al. (79)	48	49	37.82 ± 6.79	37.84 ± 6.75	4.19 ± 1.76 (Y)	4.21 ± 1.73 (Y)
Li and Huang (80)	30	30	52.7 ± 5.2	53.3 ± 5.8	3.8 ± 1.8 (Y)	3.8 ± 1.8 (Y)
Jing (81)	40	40	46.5 ± 2.0	45.9 ± 2.1	N/A	N/A
Yu et al. (82)	19	18	45.98 ± 5.08	46.38 ± 5.14	4.46 ± 1.05 (Y)	4.34 ± 1.02 (Y)
Chen et al. (83)	40	40	48 ± 14	47 ± 13	0.92 ± 0.41 (Y)	0.94 ± 0.44 (Y)
He et al. (84)	38	40	46.8 ± 11.0	43.7 ± 11.1	N/A	N/A
Ding (85)	40	40	N/A	N/A	44.17 ± 10.45 (M)	42.89 ± 14.56 (M)
Fan (86)	44	44	N/A	N/A	N/A	N/A
Gu et al. (87)	34	35	N/A	N/A	N/A	N/A
Li (88)	37	37	43.82 ± 2.08	42.49 ± 2.17	N/A	N/A
Huang and Ma (89)	40	40	48.69 ± 1.52	48.65 ± 1.57	6.38 ± 0.23 (M)	6.35 ± 0.24 (M)
Niu (90)	66	66	41.75 ± 5.06	40.29 ± 5.33	7.32 ± 2.11 (D)	7.80 ± 2.25 (D)
Zhang (91)	30	30	52.41 ± 4.09	52.38 ± 4.12	2.44 ± 0.06 (Y)	2.42 ± 0.07 (Y)
Tian (92)	30	30	43.95 ± 12.25	44.19 ± 10.95	4.18 ± 1.74 (Y)	4.06 ± 1.54 (Y)
Du (93)	75	75	42.61 ± 12.38	41.42 ± 11.69	3.92 ± 1.63 (Y)	4.21 ± 1.72 (Y)

**Table 2:** Continuation of baseline characteristics of Studies included in the network meta-analysis.

Included in the study	Intervention measures		Reported outcomes
	Experimental Group	Control group	
Cao et al. (28)	AP-COM	COM	^a, c^
Zeng et al. (29)	AP-Mox-TUI	TUI	^a, c^
Han (30)	WNM-TUI	WNM	^a, c^
Hu and Tang (31)	AP-Mox	AP	^a, c^
Yin (32)	AP-Mox	AP	^a, c^
She Ruitao and Yuanfang (33)	AP-TUI	AP	^a, c^
Miao et al. (34)	AP-COM	AP	^a, c^
Ma et al. (35)	AP-APT	AP	^a, c^
Li (36)	WNM-APT	WNM	^a, c^
Chen et al. (37)	AP-Mox	AP	^a, c^
Hu et al. (38)	WNM-TUI	TUI	^a, c^
Yue et al. (39)	AP-TUI	AP	^a, c^
Cai et al. (40)	EA-MED	MED	^a^
Wang et al. (41)	AP-MED	AP	^a, c^
Chen et al. (42)	WNM-APT	MED	^a, c^
Wan (43)	EA-MED	MED	^a, c^
Jiang et al. (44)	AP-Mox	MED	^a, c^
Chen et al. (45)	AP-TUI	TUI	^b, c^
Su (46)	WNM	TUI	^b^
Lin et al. (47)	AP-Mox	AP	^c^
Zhuang et al. (48)	EA-MOX	EA	^c^
Zhu Fuping and Shunyi (49)	EA-MOX	EA	^c^
Li Yuemei and Yanhui (50)	EA-MOX	EA	^c^
Lixia (51)	EA-MOX	EA	^c^
Wei (52)	AP-MOX	AP	^c^
Jian (53)	AP-MOX	AP	^c^
Yi et al. (54)	AP-MOX	AP	^c^
Huang and Wang (55)	AP-MOX	AP	^c^
Shen et al. (56)	AP-MOX	AP	^c^
Qin and Gu (57)	EA-MOX	EA	^c^
Gong and Wang (58)	EA-MOX	EA	^c^
Qiu (59)	EA-APT	EA	^c^
Zhan (60)	AP-COM	AP	^c^
Zhang and Zhang (61)	EA-TUI	EA	^c^
Yi (62)	AP-TUI	AP	^c^
Li (63)	EA-TUI	EA	^c^
Li Zhiqin (64)	AP-COM	AP	^c^
Fan Dehui (65)	EA-TUI	EA	^c^
Min and Zhang (66)	AP-COM	AP	^c^
Chen (67)	AP-COM	AP	^c^
Teng and Duan (68)	AP-COM	COM	^c^
Guo and He (69)	AP-COM	COM	^c^
Yu (70)	AP-COM	COM	^c^
Wu et al. (71)	AP-MED	MED	^c^
Guo and Yan (72)	AP	MED	^c^
Luo et al. (73)	EA	MED	^c^
Yu et al. (74)	AP	MED	^c^
Deng (75)	AP	MED	^c^
Lin et al. (76)	WNM	MED	^c^
Hua and Li (77)	AP	MED	^c^
Wu et al. (78)	AP	MED	^c^
Wang et al. (79)	WNM-TUI	WNM	^c^
Li and Huang (80)	AP-MED	MED	^c^
Jing (81)	AP	MED	^c^
Yu et al. (82)	AP-Mox	AP	^c^
Chen et al. (83)	EA-MOX	EA	^b, c^
He et al. (84)	AP-TUI	MED	^a, b, c^
Ding (85)	AP-MED	MED	^c^
Fan (86)	WNM-TUI	WNM	^a, b, c^
Gu et al. (87)	WNM-TUI	MED	^a, c^
Li (88)	EA	MED	^c^
Huang and Ma (89)	AP	COM	^c^
Niu (90)	AP-TUI-MED	MED	^c^
Zhang (91)	WNM	AP	^c^
Tian (92)	WNM-TUI	TUI	^a, c^
Du (93)	WNM-TUI	WNM	^c^

^a^ESCV.

^b^DHI.

^c^Efficiency.

Methodological quality of all 66 included RCTs was evaluated using the Cochrane Risk of Bias Tool 2.0 (RoB 2). Among these, seven studies were assessed as having a low overall risk of bias, whereas 59 studies were judged to present some concerns regarding potential bias.

**Figure 2:**
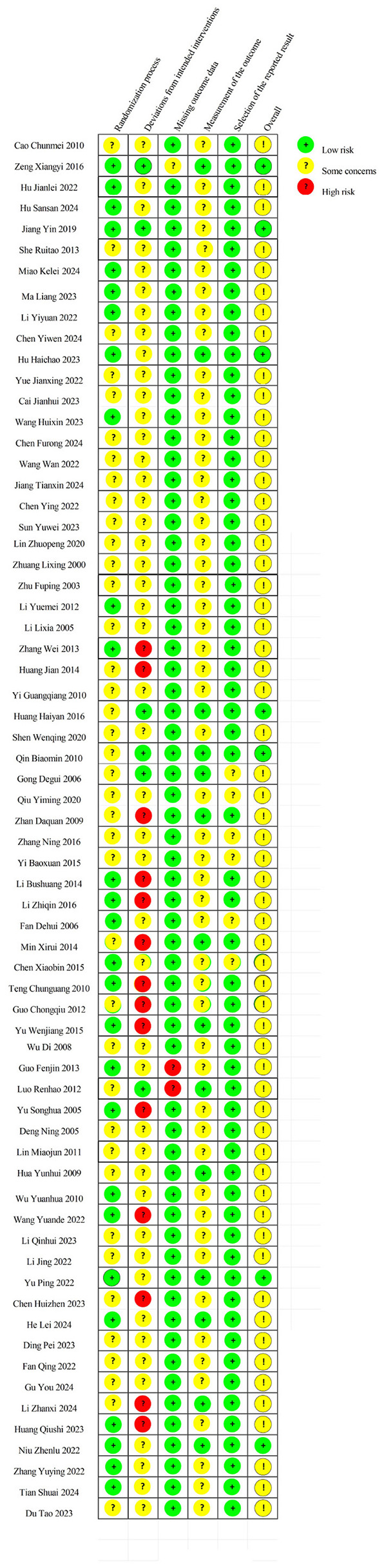
Summary of risk of bias assessment using the cochrane risk of bias 2.0 (RoB 2) tool for all included RCTs.

A detailed breakdown of the risk of bias assessment across the five RoB 2 domains is provided in Figure 2, which illustrates domain-specific judgments for each included study and an overall summary of bias distribution.

### 3.2 Network meta-analyses

#### 3.2.1 Comparisons of overall effectiveness rate

**Figure 3:**
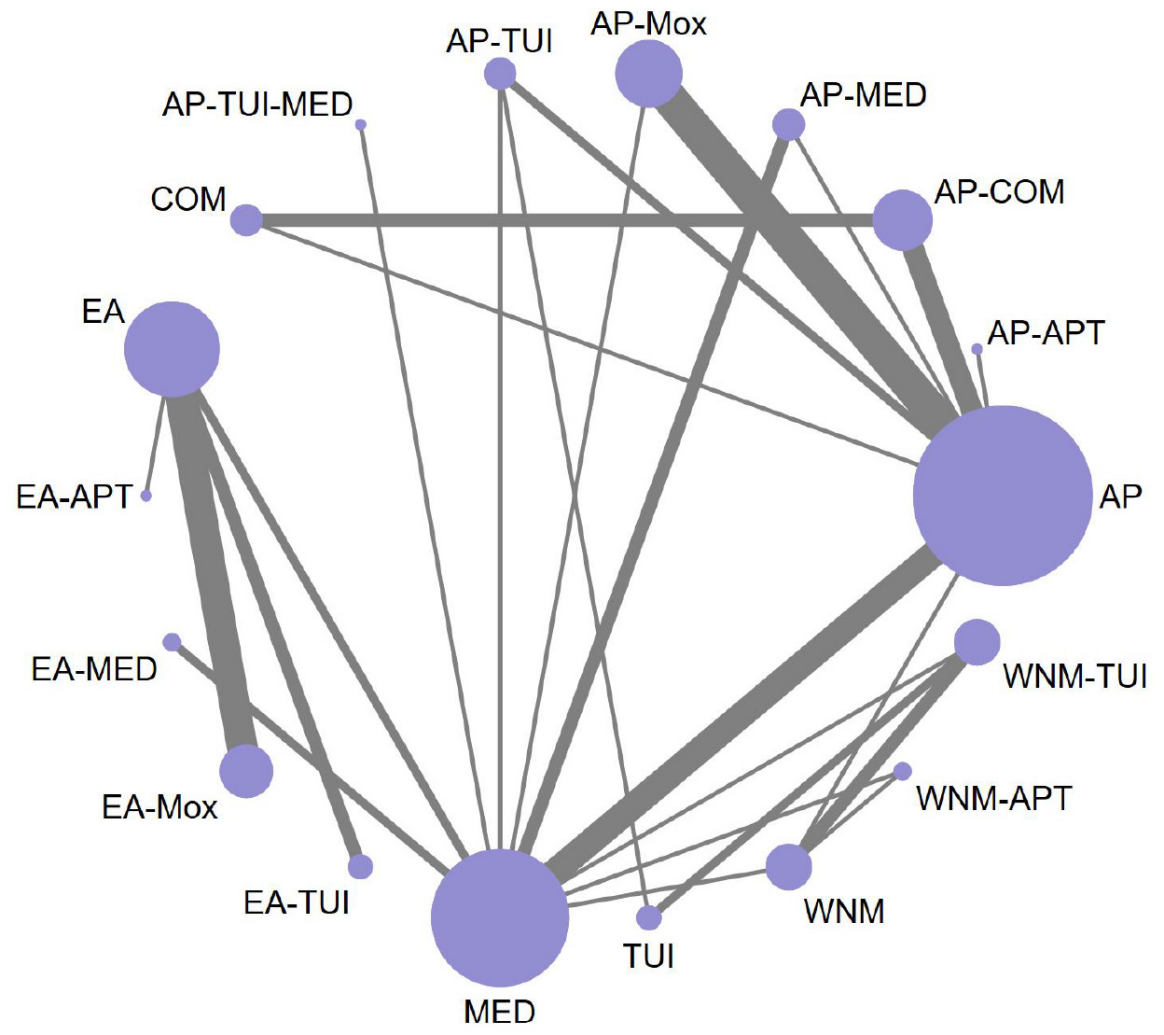
Network diagram of pairwise comparisons for overall effectiveness rate.

A total of 47 RCTs reported the overall effectiveness rate in patients with cervical vertigo. The network meta-analysis incorporated 18 distinct intervention strategies. The geometry of the treatment comparisons is visualized in Figure 3, which depicts the structure and interconnections among all included interventions.

The resulting network diagram contained several closed loops, which enabled formal inconsistency testing. The global inconsistency test yielded a *P*-value ≥ 0.05, indicating no statistically significant inconsistency; therefore, the consistency model was adopted for the analysis. Loop-specific inconsistency tests also showed that the 95% confidence intervals (CIs) of the inconsistency factors included zero, suggesting minimal inconsistency and favorable homogeneity. Overall, both direct and indirect comparisons produced consistent results.

The odds ratios (ORs) and corresponding 95% confidence intervals (CIs) revealed the following findings:

Compared with EA–MED, both EA–TUI (OR = 1.29; 95% CI: 1.06–1.58) and EA–MOX (OR = 1.28; 95% CI: 1.06–1.56) showed higher overall effectiveness rates. Among monotherapies, EA was more effective than TUI (OR = 1.36; 95% CI: 1.12–1.67), and AP also outperformed TUI (OR = 1.19; 95% CI: 1.02–1.39). In addition, EA showed a modest advantage over AP (OR = 1.14; 95% CI: 1.00–1.31).

Among monotherapies, EA was more clinically effective than TUI (OR = 1.36; 95% CI: 1.12–1.67). Similarly, AP also outperformed TUI (OR = 1.19; 95% CI: 1.02–1.39). In addition, EA showed greater effectiveness than AP (OR = 1.14; 95% CI: 1.00–1.31), suggesting a modest advantage of EA over AP among monotherapies.

When combination therapies were compared with monotherapies, EA–TUI demonstrated the largest benefit relative to TUI alone (OR = 1.53; 95% CI: 1.23–1.89). Similarly, EA–MOX (OR = 1.52; 95% CI: 1.23–1.87) and EA–APT (OR = 1.50; 95% CI: 1.17–1.92) were also more effective than TUI, indicating potentially favorable options for cervical vertigo.

According to the ranking based on effectiveness in improving the total effectiveness rate , the top three intervention strategies were as follows: EA–TUI > EA–MOX > EA–APT.

**Figure 4:**
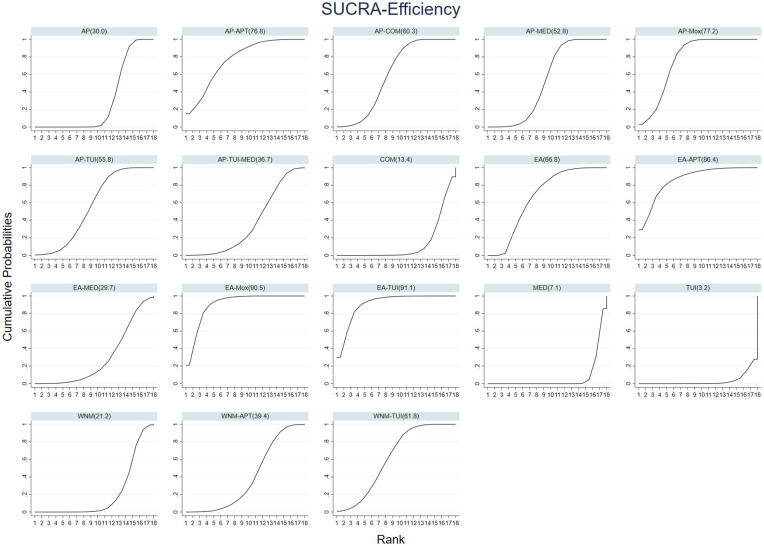
Cumulative ranking probability curves and SUCRA values for the total effectiveness rate across 18 acupuncture- and tuina-based interventions.

**Figure 5:**
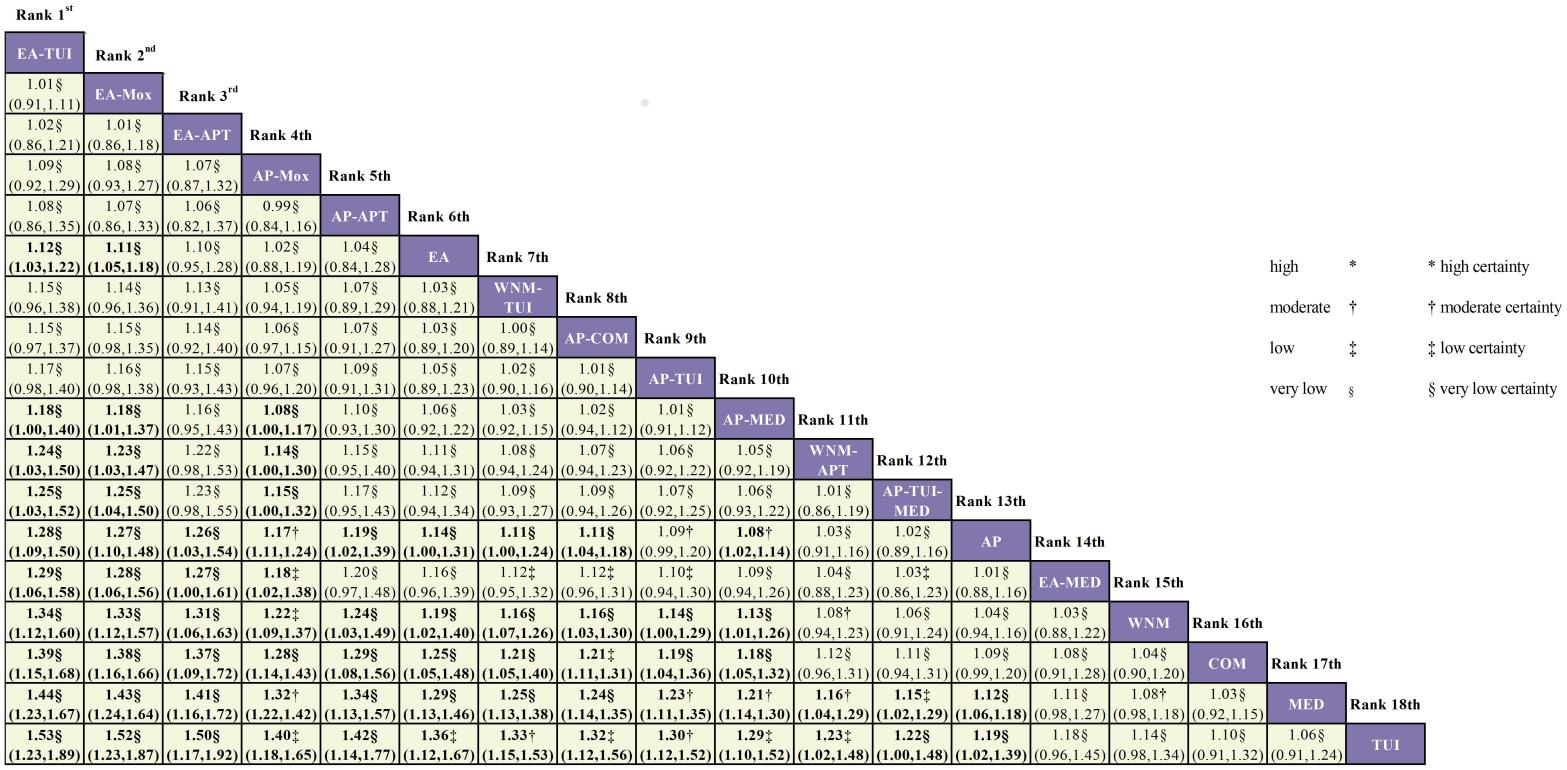
League table of pairwise comparisons for the total effectiveness rate across acupuncture- and tuina-related interventions (OR, 95% CI), with certainty of evidence assessed using CINeMA. ^*^High certainty; ^†^moderate certainty; ^‡^low certainty; ^§^very low certainty.

The cumulative ranking plot is presented in Figure 4, and detailed pairwise comparison results are shown in Figure 5. This league table summarizes the pairwise ORs and 95% CIs for the overall effectiveness rate among 18 different acupuncture and Tuina interventions for cervical vertigo.

Interventions are ordered from the top-left (more favorable) to the bottom-right (less favorable) according to SUCRA values. Each cell reports the odds ratio (OR) and 95% confidence interval (CI) for the intervention in the column compared with the intervention in the row. Boldface indicates statistical significance (i.e., the 95% CI does not include 1).

##### 3.2.1.1 Meta-regression for the total effectiveness rate

A network meta-regression was performed to examine whether heterogeneity in the definition of the total effectiveness rate (coded as an ordinal covariate with nine categories, cov1 = 1–9) modified treatment effects results are presented in Table S2 in Data Sheet 1 Overall, the threshold definition was not significantly associated with effect estimates across most comparisons (*e*.*g*._,_*y*_*C*_*P* = 0.611_;_*y*_*D*_*P* = 0.659_;_*y*_*E*_*P* = 0.408_;_*y*_*F*_*P* = 0.273 _;_*y*_*N*_*P* = 0.611_;_*y*_*R*_*P* = 0.333), suggesting that variability in response criteria was unlikely to be a major driver of the primary findings. A borderline signal was observed in one comparison (_y_H β = −0.436, *P* = 0.060), whereas several nodes yielded extremely imprecise estimates with very wide confidence intervals (*e*.*g*._,_*y*_*I*__,_*y*_*L*__,_*y*_*M*_), indicating sparse data.

#### 3.2.2 Comparisons of ESCV

**Figure 6:**
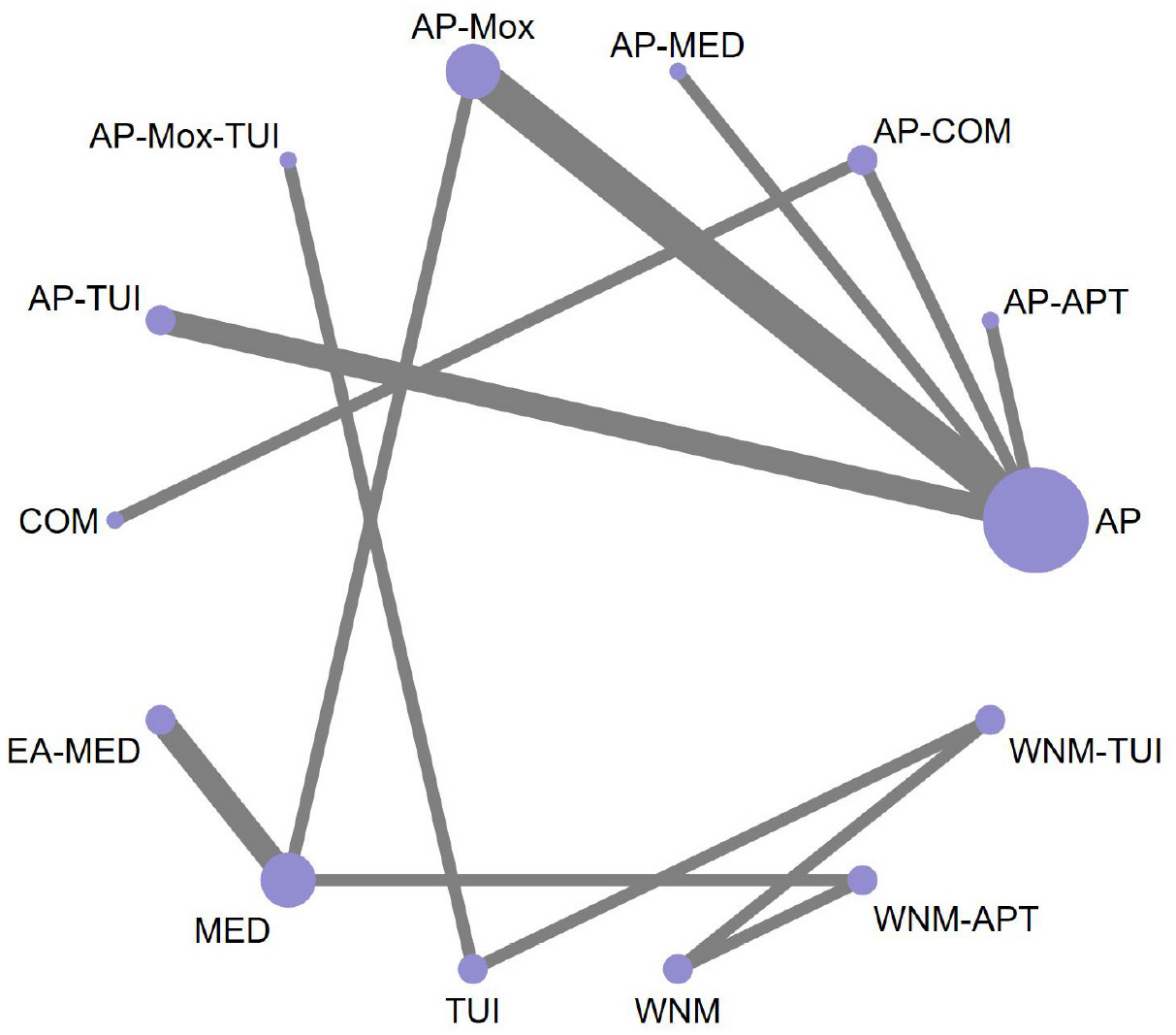
Network diagram of pairwise comparisons for ESCV.

A total of seventeen RCTs reported outcomes based on the ESCV, involving fourteen distinct intervention strategies. The network geometry of these acupuncture- and Tuina-related interventions is presented in Figure 6.

As the ESCV network structure did not form any closed loops, a consistency model was applied for analysis. Based on the mean differences (MDs) and their corresponding 95% confidence intervals (CIs), the following findings were observed:

Among combination therapies, AP–COM was significantly more effective than AP–MOX–TUI in improving ESCV (MD = 6.52; 95% CI: 1.48–11.57). Similarly, AP–MOX also significantly outperformed AP–MOX–TUI (MD = 6.34; 95% CI: 1.64–11.03), indicating superior efficacy.

Among monotherapies, AP achieved significantly greater improvement in ESCV than TUI (MD = 8.38; 95% CI: 4.66–12.11), suggesting a more favorable therapeutic effect.

**Figure 7:**
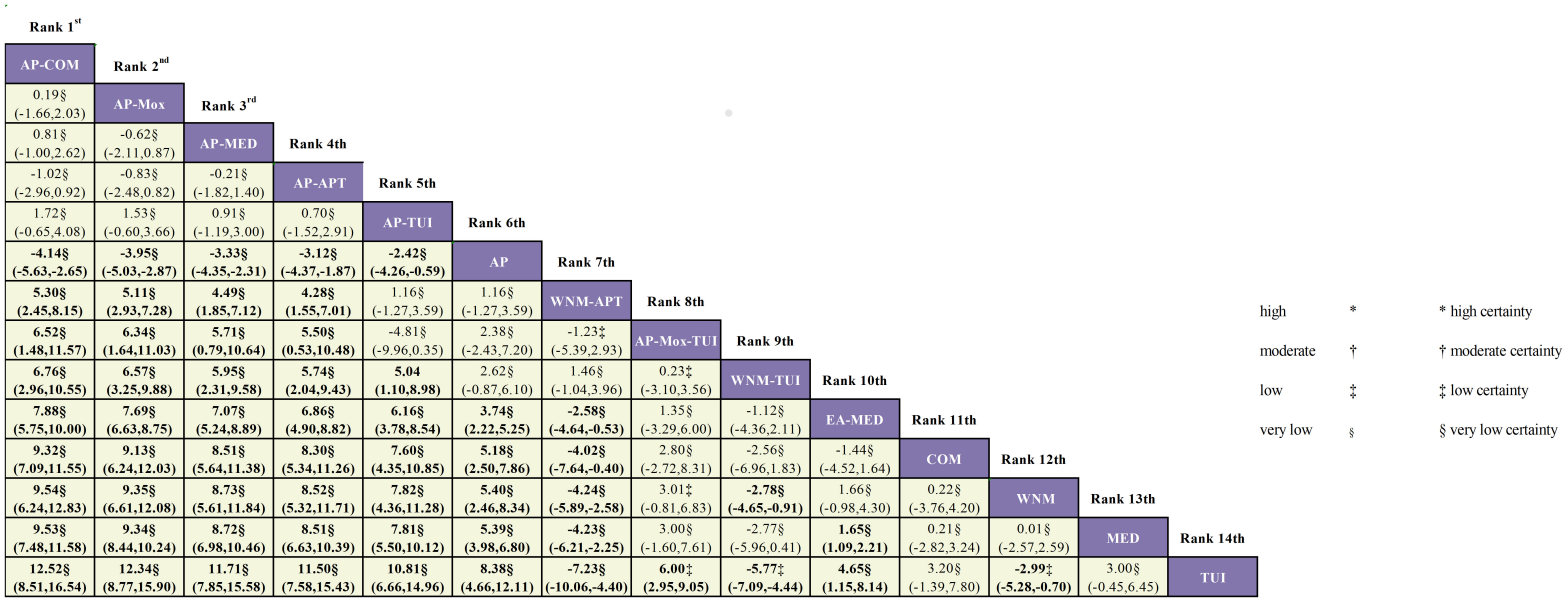
League table of pairwise comparisons for ESCV across acupuncture- and Tuina-related interventions (MD, 95% CI), with certainty of evidence assessed using CINeMA. ^*^High certainty; ^†^moderate certainty; ^‡^low certainty; ^§^very low certainty.

When comparing combination therapies with a single therapy, AP–COM (MD = 12.52; 95% CI: 8.51–16.54), AP–MOX (MD = 12.34; 95% CI: 8.77–15.90), and AP–MED (MD = 11.71; 95% CI: 7.85–15.58) all significantly outperformed TUI alone in improving ESCV. Detailed pairwise comparison results are presented in Figure 7. This league table presents the MDs and their corresponding 95% CIs for the effectiveness of 14 Acupuncture- and Tuina-based interventions in improving ESCV. Interventions are ranked from left to right and top to bottom in descending order of overall efficacy (based on cumulative probability or SUCRA ranking). Each cell displays the MD (95% CI) of the intervention listed in the column compared to the intervention listed in the row. Bolded results denote statistically significant differences (i.e., 95% CI does not include 0).

**Figure 8:**
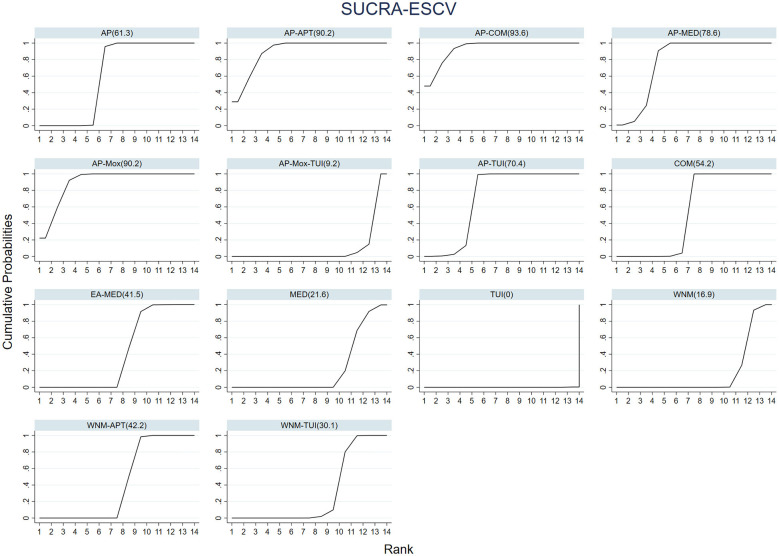
Cumulative probability ranking curves for ESCV improvement across 14 acupuncture and tuina-based interventions.

In terms of treatment ranking for improving ESCV, the top three interventions were:

AP–COM > AP–MOX > AP–MED, as illustrated in Figure 8.

#### 3.2.3 Comparisons of DHI

**Figure 9:**
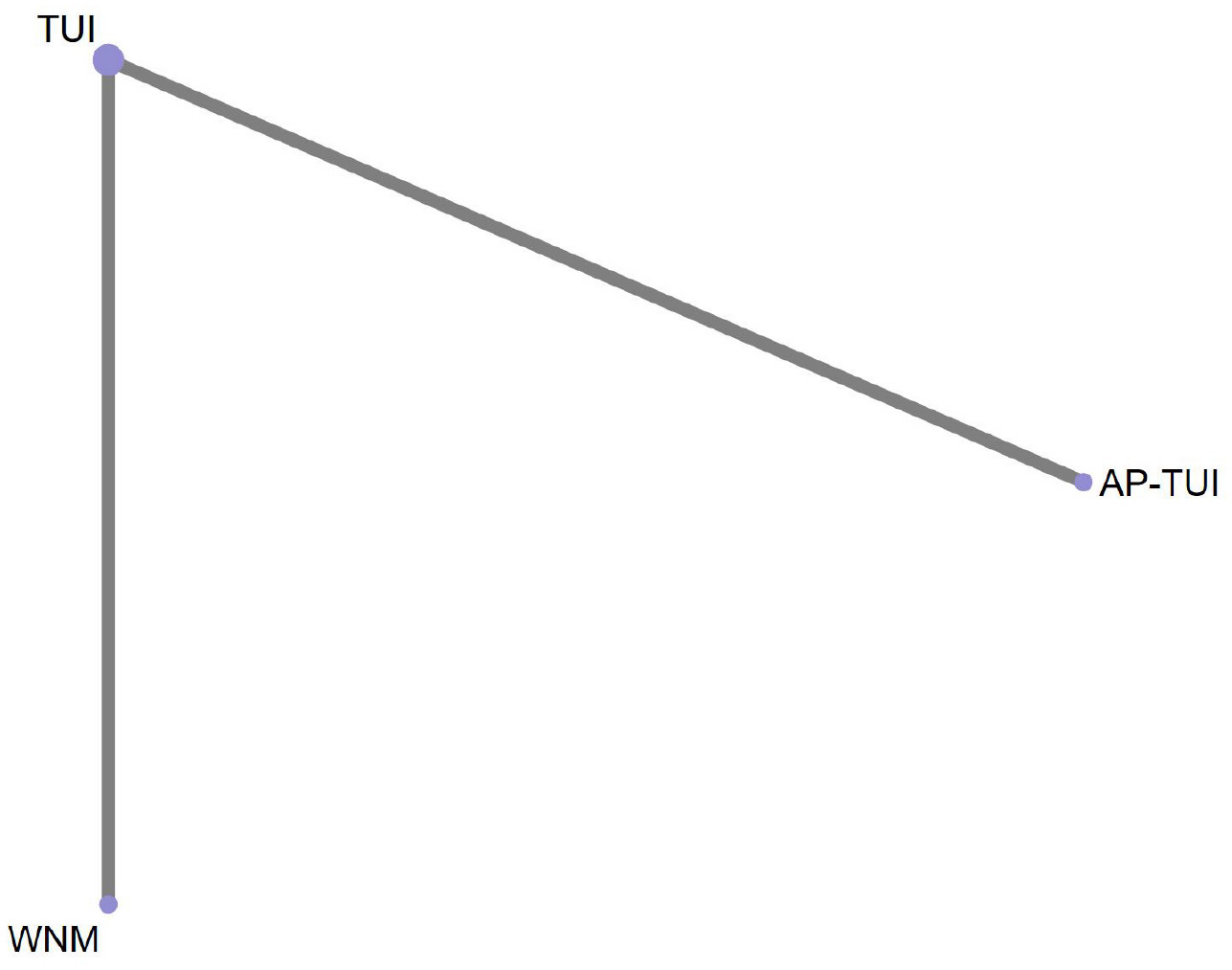
Network diagram of pairwise comparisons for DHI.

A total of two RCTs reported outcomes based on the Dizziness Handicap Inventory (DHI), involving three distinct intervention strategies. The network geometry of these interventions is presented in Figure 9.

As the network structure did not form any closed loops, a consistency model was applied for analysis. Based on the standardized mean differences (SMDs) and their corresponding 95% confidence intervals (CIs), the following finding was observed:

Among monotherapies, warm needle moxibustion (WNM) was significantly more effective than tuina (TUI) in reducing DHI scores (SMD = 2.04; 95% CI: 1.49–2.58), indicating superior efficacy in alleviating dizziness-related disability.

**Figure 10:**
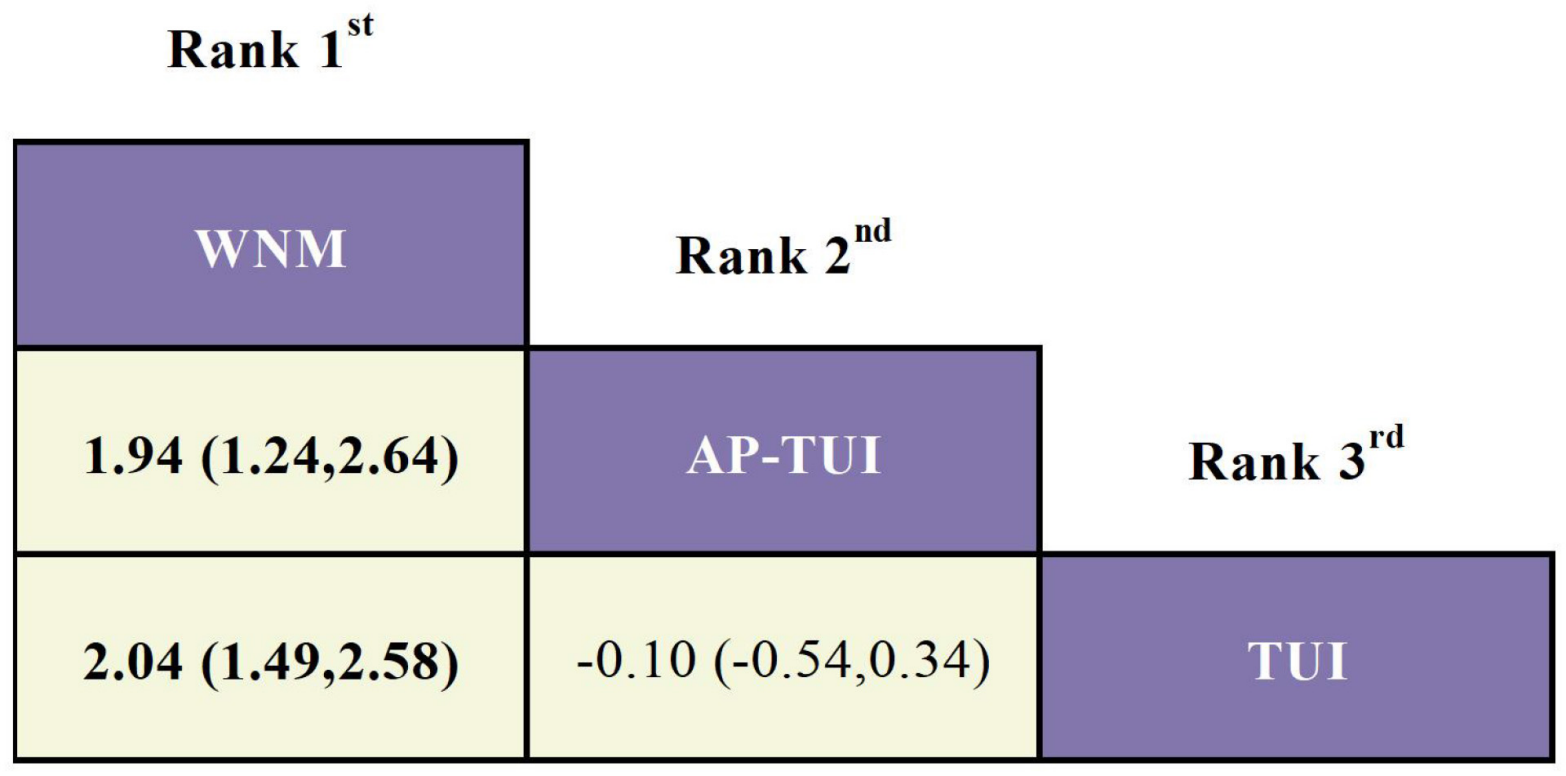
League table of pairwise comparisons among three acupuncture and tuina-based interventions for improving DHI scores (SMD, 95% CI). Interventions are ranked from top-left (best) to bottom-right (worst) based on their estimated clinical effectiveness in lowering DHI scores.

When comparing combination therapy with monotherapy, AP–TUI significantly outperformed WNM in reducing DHI scores (SMD = 1.94; 95% CI: 1.24–2.64), indicating a more favorable therapeutic outcome. Detailed pairwise comparison results are presented in Figure 10. Interventions are ranked from top-left (best) to bottom-right (worst) based on their estimated clinical effectiveness in lowering DHI scores.

**Figure 11:**
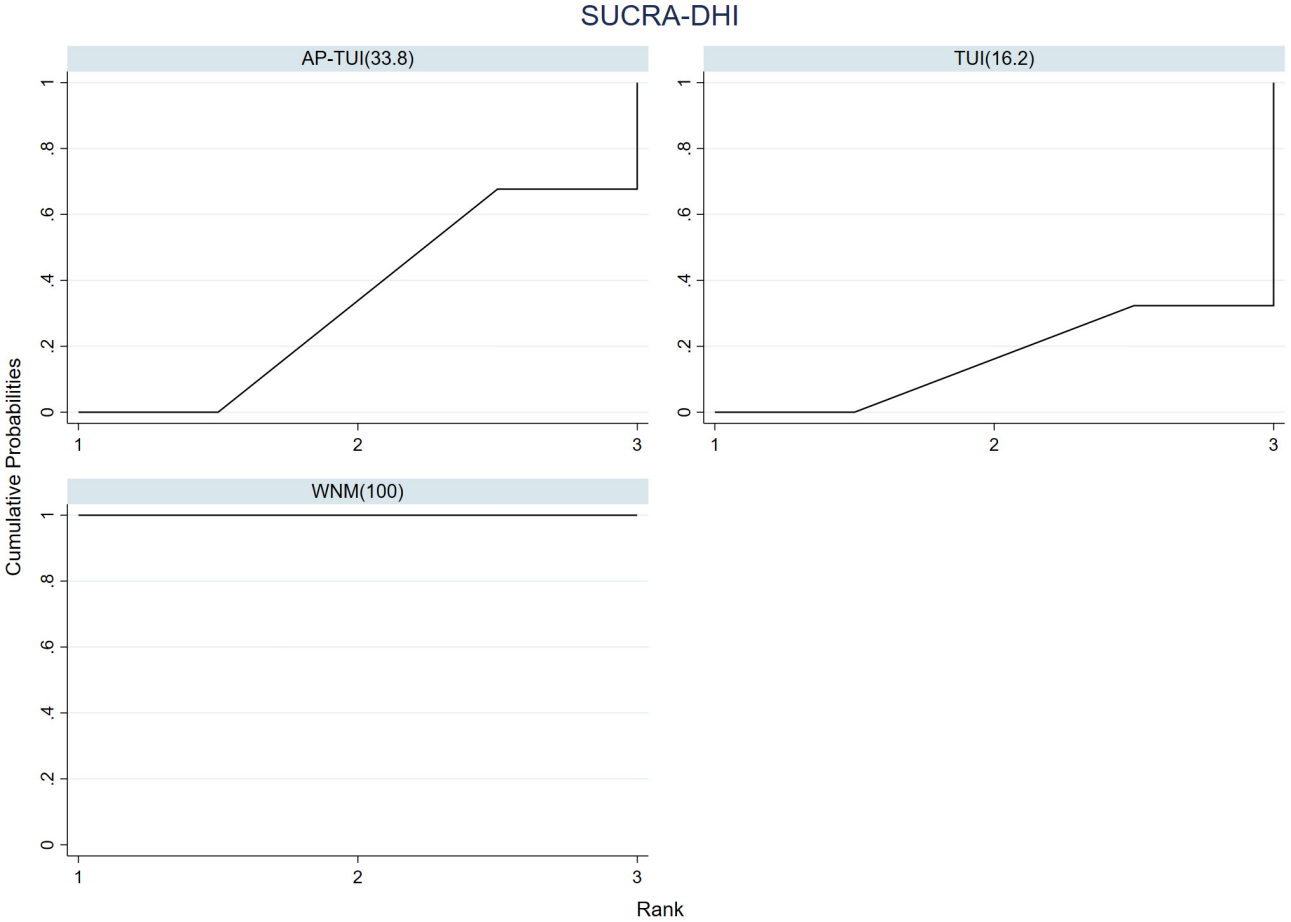
Cumulative ranking probability curves and SUCRA values for DHI improvement across three acupuncture- and tuina-based interventions.

In terms of treatment ranking for reducing DHI scores, the top three interventions were: WNM > AP–TUI > TUI, as illustrated in Figure 11.

### 3.3 Assessment of publication bias

Funnel plots were generated for the primary and secondary outcomes—including overall effectiveness rate, ESCV, and DHI—to evaluate the presence of potential publication bias.

**Figure 12:**
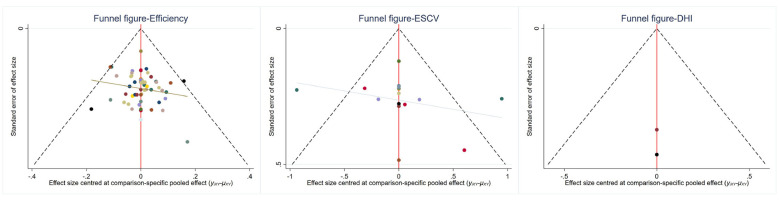
Funnel plots for overall effectiveness rate, ESCV, and DHI.

The funnel plots exhibited a symmetrical distribution of study data points, with no evident asymmetry or extreme outliers. These findings suggest a low risk of publication bias among the included studies. The funnel plots are presented in Figure 12.

### 3.4 Sensitivity analysis

To examine whether methodological differences and risk of bias influenced the network meta-analysis, we performed a sensitivity analysis. Because no trials contributing to the ESCV or DHI outcomes were rated as high risk by RoB 2, this exclusion-based sensitivity analysis was not applicable to those endpoints; therefore, it was conducted only for the total effectiveness rate. High–risk–of–bias studies (RoB 2) were excluded, and the network meta-analysis was re-run, with the updated league table presented in Figure S1 in Data Sheet 1.

Overall, the findings for the total effectiveness rate showed some sensitivity to study quality. Compared with the primary analysis (Figure 5), the network remained broadly comparable, although the number of intervention nodes decreased from 18 to 17 due to the removal of the COM node, and effect estimates generally moved toward the null (OR ≈ 1.0) with wider confidence intervals. In terms of ranking, the primary analysis suggested EA-TUI > EA-MOX > EA-APT, whereas after excluding high-risk studies, the leading hierarchy shifted to AP-COM > AP-APT > AP-Mox; correspondingly, EA-TUI, EA- MOX, and EA-APT dropped to ranks 4–6, indicating that identification of the “best” intervention is partly contingent on higher-risk evidence. Consistent with this, the blue cells in Figure S1 in Data Sheet 1 highlight comparisons that changed from statistically significant to non-significant after exclusion (i.e., estimates attenuated toward 1.0 and 95% CIs crossed 1), mainly involving AP- vs. EA-based combination regimens and several mid-ranked combination strategies—for example, AP vs. EA-TUI (OR 1.20, 95% CI 0.98–1.47), AP vs. EA-MOX (OR 1.19, 95% CI 0.99–1.44), AP vs. EA-APT (OR 1.19, 95% CI 0.95–1.50), AP-TUI vs. AP-Mox (OR 0.93, 95% CI 0.85–1.01), AP-MED vs. AP-Mox (OR 1.12, 95% CI 0.97–1.29), and EA-MED vs. EA-TUI (OR 0.86, 95% CI 0.71–1.04). Taken together, while the overall direction of effects was largely consistent (combination strategies still tended to rank highly), shifts in several relative effects and rankings suggest that the robustness of the total effectiveness rate results should be interpreted with caution.

Accordingly, SUCRA-based rankings should be viewed as hypothesis-generating rather than definitive.

### 3.5 GRADE analysis and the credibility of the evidence

We rated concerns within each domain as “No concerns,” “Some concerns,” or “Major concerns.” Following GRADE guidance, we downgraded the evidence by one level for “Some concerns” and by two levels for “Major concerns,” finally classifying the overall certainty as High, Moderate, Low, or Very Low.

Applying the CINeMA framework revealed a varied distribution of evidence certainty across outcomes. For the ESCV, among 91 comparisons, we rated 6 as “Low” and 85 as “Very Low” (Figure 7). For the total effectiveness rate, among 154 comparisons, we rated 11 as “Moderate,” 21 as “Low,” and 121 as “Very Low” (Figure 5). We did not perform a formal certainty assessment for the DHI due to insufficient data for a meaningful network comparison.

## 4 Discussion

### 4.1 Principal findings

This network meta-analysis compared 18 acupuncture- and manual-therapy–related strategies for cervical vertigo across overall effectiveness, ESCV, and DHI. Overall, combination regimens tended to rank higher than monotherapies, although the certainty of evidence was generally low. For overall effectiveness, EA–TUI showed the most favorable performance, with EA also ranking highly among monotherapies. For ESCV, AP–COM was among the most effective options, whereas TUI consistently ranked lower. For DHI, evidence was sparse; WNM appeared to yield the most significant reduction in disability, but conclusions are limited by the small number of contributing trials. Sensitivity analyses suggested that some estimates were unstable when high-risk studies were excluded, and the findings should therefore be interpreted cautiously.

### 4.2 Mechanistic interpretation

Although numerous RCTs have reported benefits of acupuncture- and manual-therapy–related interventions for cervical vertigo, the evidence base remains heterogeneous and direct head-to-head comparisons across multiple competing strategies are limited, which makes pairwise approaches less informative for comparative decision-making (21–23). A plausible explanation for the apparent advantage of combination regimens is their multi-component, multi-target nature. Needle-based stimulation (EA/AP) may modulate nociceptive processing and autonomic symptoms, while Tuina/osteopathic manipulation may improve cervical soft-tissue tension, joint mobility, and proprioceptive input, which are frequently implicated in cervicogenic dizziness. Moxibustion or warm-needle techniques may provide thermal stimulation that facilitates muscle relaxation and local circulation. Prior work also suggests that electroacupuncture may act through multi-target pathways involving modulation of vertebral artery blood flow, cervical muscle tone, and autonomic nervous system function (24). However, given the overall low certainty of evidence and sensitivity to risk-of-bias assumptions, these mechanistic interpretations should be considered hypothesis-generating.

Notably, combination therapies did not uniformly demonstrate superior performance. For example, AP–MOX–TUI ranked relatively low for ESCV improvement, and AP–TUI–MED showed only modest effects for overall effectiveness; similar patterns were observed for DHI. These findings challenge the assumption that adding more components necessarily improves outcomes. One plausible explanation is a dose–response ceiling effect proposed in related literature, whereby additional components beyond a therapeutic threshold may yield limited incremental benefit or introduce negative interactions (25, 26). Once a therapeutic threshold is reached, treatment effects may approach an optimal plateau; further intensification may not yield additional benefit and may even have adverse consequences.

### 4.3 ESCV and clinical/rehabilitation relevance

ESCV is intended to reflect overall CV severity and cervical functional impairment, capturing domains directly relevant to rehabilitation and functional recovery; compared with an overall “effectiveness rate,” it may better reflect functional change and symptom impact. In our network, AP–COM showed the greatest improvement in ESCV, followed by AP–APT and AP–MOX, and AP also outperformed TUI among monotherapies. These findings are broadly consistent with previous meta-analyses reporting that acupuncture can reduce the severity of vertigo-related symptoms in CV (27). From a rehabilitation perspective, greater ESCV improvement may indicate not only symptom reduction but also better neck-related function, which may translate into improved activity tolerance and daily functioning.

For DHI, WNM appeared to yield the largest reduction in dizziness-related disability compared with TUI and AP–TUI. Because DHI data were limited, these results should be interpreted cautiously; nevertheless, they suggest that symptom-specific modalities may confer targeted benefits for disability-related outcomes in some patients.

### 4.4 Clinical implications, limitations, and future research

Our findings may help clinicians align treatment selection with specific clinical objectives (e.g., symptom relief vs. functional recovery), while recognizing uncertainty in the underlying evidence. A major gap remains the lack of well-designed, head-to-head trials directly comparing clearly defined acupuncture and manual-therapy protocols. Future RCTs should standardize diagnostic criteria, intervention dose (frequency/duration), and outcome reporting, and should include adequate follow-up and transparent adverse-event reporting to strengthen confidence in comparative estimates.

Several limitations warrant consideration. First, some included RCTs provided insufficient detail regarding randomization methods, which may increase the risk of bias and reduce internal validity. Second, substantial clinical heterogeneity existed even within the same labeled intervention (e.g., variation in acupoint selection, manipulation techniques, treatment frequency, and duration), which may compromise comparability across trials. Third, most participants were Chinese, limiting generalizability to other populations and healthcare settings. Despite these limitations, the available evidence supports potential benefits of acupuncture- and Tuina-related approaches for CV. Future studies should prioritize methodological rigor (e.g., allocation concealment, prespecified outcomes, and CONSORT-compliant reporting) and conduct direct comparative trials to complement indirect evidence and improve the certainty and applicability of findings.

## Data Availability

The original contributions presented in the study are included in the article/Supplementary material, further inquiries can be directed to the corresponding author.
