# Supplementary material for: Comparative effectiveness of acupuncture and Tuina for cervical vertigo: a systematic review and network meta-analysis of randomized controlled trials
Source: Front Neurol. 2026 May 8;17:1691312. doi: 10.3389/fneur.2026.1691312 (PMC13193925; doi:10.3389/fneur.2026.1691312)
Supplement: Supplementary file 1 [file Data_Sheet_1.pdf]

Supplementary Table S1. Operational definitions of the total effectiveness rate across trials and coding scheme (A–I).

| Coding | Categories (trial-defined response levels) | Formula (rate, %) |
|---|---|---|
| A | cured; markedly effective; effective; ineffective | (cured + markedly effective + effective) / total × 100 |
| B | cured; markedly effective; improved; ineffective | (cured + markedly effective) / total × 100 |
| C | cured; markedly effective; improved; ineffective | (cured + markedly effective + improved) / total × 100 |
| D | cured; effective; ineffective | (cured + effective) / total × 100 |
| E | cured; markedly effective; effective; ineffective | (cured) / total × 100 |
| F | markedly effective; effective; ineffective | (markedly effective + effective) / total × 100 |
| G | cured; improved; ineffective | (cured + improved) / total × 100 |
| H | cured; markedly effective; ineffective | (cured + markedly effective) / total × 100 |
| I | clinical control; markedly effective; effective; ineffective | (clinical control + markedly effective + effective) / total × 100 |

Supplementary Table S2.Meta-regression for the total effectiveness rate

| | | Coefficient | Std. err | z | P>\|Z\| | 95% conf. interval | |
|---|---|---|---|---|---|---|---|
| _y_B | | | | | | | |
| | _cons | 1.677 | 0.813 | 2.060 | 0.039 | 0.084 | 3.271 |
| _y_C | | | | | | | |
| | cov1 | -0.130 | 0.256 | -0.510 | 0.611 | -0.633 | 0.372 |
| | _cons | 1.406 | 0.704 | 2.000 | 0.046 | 0.026 | 2.786 |
| _y_D | | | | | | | |
| | cov1 | -0.178 | 0.403 | -0.440 | 0.659 | -0.968 | 0.612 |
| | _cons | 1.418 | 0.697 | 2.030 | 0.042 | 0.052 | 2.785 |
| _y_E | | | | | | | |
| | cov1 | 0.249 | 0.301 | 0.830 | 0.408 | -0.341 | 0.839 |
| | _cons | 0.990 | 0.494 | 2.000 | 0.045 | 0.022 | 1.958 |
| _y_F | | | | | | | |
| | cov1 | -0.189 | 0.173 | -1.100 | 0.273 | -0.528 | 0.149 |
| | _cons | 1.101 | 0.459 | 2.400 | 0.016 | 0.202 | 2.001 |
| _y_G | | | | | | | |
| | _cons | 0.004 | 0.870 | 0.000 | 0.996 | -1.702 | 1.710 |
| _y_H | | | | | | | |
| | cov1 | -0.436 | 0.212 | -2.050 | 0.060 | -0.852 | -0.019 |
| | _cons | 0.610 | 0.802 | 0.760 | 0.447 | -0.962 | 2.182 |
| _y_I | cov1 | 0.452 | 7.781 | 0.060 | 0.954 | -14.799 | 15.703 |

| | | | | | | | |
|---|---|---|---|---|---|---|---|
| | _cons | -1.296 | 46.670 | -0.030 | 0.978 | -92.768 | 90.176 |
| _y_J | | | | | | | |
| | _cons | -0.036 | 38.896 | 0.000 | 0.999 | -76.270 | 76.198 |
| _y_K | | | | | | | |
| | cov1 | -0.134 | 0.152 | -0.880 | 0.377 | -0.432 | 0.164 |
| | _cons | -0.012 | 0.736 | -0.020 | 0.987 | -1.454 | 1.430 |
| _y_L | | | | | | | |
| | cov1 | 0.192 | 7.796 | 0.020 | 0.980 | -15.087 | 15.471 |
| | _cons | 0.635 | 46.686 | 0.010 | 0.989 | -90.868 | 92.138 |
| _y_M | | | | | | | |
| | cov1 | -0.885 | 7.853 | -0.110 | 0.910 | -16.277 | 14.507 |
| | _cons | 1.684 | 46.704 | 0.040 | 0.971 | -89.855 | 93.222 |
| _y_N | | | | | | | |
| | cov1 | -0.052 | 0.102 | -0.510 | 0.611 | -0.252 | 0.148 |
| | _cons | -1.015 | 0.414 | -2.450 | 0.014 | -1.826 | -0.204 |
| _y_O | | | | | | | |
| | cov1 | 0.339 | 0.265 | 1.280 | 0.202 | -0.182 | 0.859 |
| | _cons | -1.179 | 0.792 | -1.490 | 0.137 | -2.732 | 0.375 |
| _y_P | | | | | | | |
| | cov1 | 0.328 | 0.179 | 1.830 | 0.067 | -0.023 | 0.680 |
| | _cons | -0.645 | 0.681 | -0.950 | 0.343 | -1.980 | 0.689 |
| _y_Q | | | | | | | |
| | _cons | 1.382 | 0.984 | 1.400 | 0.160 | -0.546 | 3.310 |
| _y_R | | | | | | | |
| | cov1 | 0.206 | 0.213 | 0.970 | 0.333 | -0.211 | 0.624 |
| | _cons | 1.035 | 0.711 | 1.450 | 0.146 | -0.359 | 2.429 |

Supplementary Table S3. CINeMA-based certainty of evidence (GRADE ratings) for the total effectiveness rate.

| Comparison | Number of studies | Within-study bias | Reporting bias | Indirectness | Imprecision | Heterogeneity | Incoherence | Confidence rating | Reason(s) for downgrading |
|---|---|---|---|---|---|---|---|---|---|
| AP:AP-APT | 1 | Some concerns | Low risk | No concerns | No concerns | No concerns | Major concerns | Very low | ["Within-study bias","Incoherence"] |
| AP:AP-COM | 5 | Major concerns | Low risk | No concerns | No concerns | No concerns | Major concerns | Very low | ["Within-study bias","Incoherence"] |
| AP:AP-MED | 1 | Some concerns | Low risk | No concerns | No concerns | No concerns | No concerns | Moderate | ["Within-study bias"] |
| AP:AP-Mox | 8 | Some concerns | Low risk | No concerns | No concerns | No concerns | No concerns | Moderate | ["Within-study bias"] |
| AP:AP-TUI | 2 | Some concerns | Low risk | No concerns | No concerns | No concerns | No concerns | Moderate | ["Within-study bias"] |
| AP:COM | 1 | Major concerns | Low risk | No concerns | Some concerns | No concerns | Major concerns | Very low | ["Within-study bias","Imprecision","Incoherence"] |
| AP:MED | 6 | Some concerns | Low risk | No concerns | No concerns | No concerns | Major concerns | Very low | ["Within-study bias","Incoherence"] |
| AP:WNM | 1 | Some concerns | Low risk | No concerns | Major concerns | No concerns | Some concerns | Very low | ["Within-study bias","Imprecision","Incoherence"] |
| AP-COM:COM | 3 | Major concerns | Low risk | No concerns | No concerns | No concerns | No concerns | low | ["Within-study bias"] |

| Comparison | Number of studies | Within-study bias | Reporting bias | Indirectness | Imprecision | Heterogeneity | Incoherence | Confidence rating | Reason(s) for downgrading |
|---|---|---|---|---|---|---|---|---|---|
| AP-MED:MED | 3 | Some concerns | Low risk | No concerns | No concerns | No concerns | No concerns | Moderate | ["Within-study bias"] |
| AP-Mox:MED | 1 | Some concerns | Low risk | No concerns | No concerns | No concerns | No concerns | Moderate | ["Within-study bias"] |
| AP-TUI:MED | 1 | Some concerns | Low risk | No concerns | No concerns | No concerns | No concerns | Moderate | ["Within-study bias"] |
| AP-TUI:TUI | 1 | Some concerns | Low risk | No concerns | No concerns | No concerns | No concerns | Moderate | ["Within-study bias"] |
| AP-TUI-MED:MED | 1 | No concerns | Low risk | No concerns | No concerns | No concerns | Major concerns | low | ["Incoherence"] |
| EA:EA-APT | 1 | Some concerns | Low risk | No concerns | Major concerns | No concerns | Major concerns | Very low | ["Within-study bias","Imprecision","Incoherence"] |
| EA:EA-Mox | 7 | Some concerns | Low risk | No concerns | No concerns | No concerns | Major concerns | Very low | ["Within-study bias","Incoherence"] |
| EA:EA-TUI | 3 | Some concerns | Low risk | No concerns | Some concerns | Some concerns | Major concerns | Very low | ["Within-study bias","Imprecision","Heterogeneity","Incoheren[illegible] |
| EA:MED | 2 | Major concerns | Low risk | No concerns | No concerns | No concerns | Major concerns | Very low | ["Within-study bias","Incoherence"] |
| EA-MED:MED | 2 | Some concerns | Low risk | No concerns | Some concerns | No concerns | Major concerns | Very low | ["Within-study bias","Imprecision","Incoherence"] |
| MED:WNM | 1 | Some concerns | Low risk | No concerns | No concerns | No concerns | No concerns | Moderate | ["Within-study bias"] |

| Comparison | Number of studies | Within-study bias | Reporting bias | Indirectness | Imprecision | Heterogeneity | Incoherence | Confidence rating | Reason(s) for downgrading |
|---|---|---|---|---|---|---|---|---|---|
| MED:WNM-APT | 1 | Some concerns | Low risk | No concerns | No concerns | No concerns | No concerns | Moderate | ["Within-study bias"] |
| MED:WNM-TUI | 1 | Some concerns | Low risk | No concerns | No concerns | No concerns | Major concerns | Very low | ["Within-study bias","Incoherence"] |
| TUI:WNM-TUI | 2 | Some concerns | Low risk | No concerns | No concerns | No concerns | No concerns | Moderate | ["Within-study bias"] |
| WNM:WNM-APT | 1 | Some concerns | Low risk | No concerns | No concerns | No concerns | No concerns | Moderate | ["Within-study bias"] |
| WNM:WNM-TUI | 3 | Some concerns | Low risk | No concerns | No concerns | No concerns | Major concerns | Very low | ["Within-study bias"] |
| AP:AP-TUI-MED | 0 | Some concerns | Low risk | No concerns | Major concerns | No concerns | Major concerns | Very low | ["Within-study bias","Imprecision","Incoherence"] |
| AP:EA | 0 | Major concerns | Low risk | No concerns | Some concerns | No concerns | Major concerns | Very low | ["Within-study bias","Imprecision","Incoherence"] |
| AP:EA-APT | 0 | Some concerns | Low risk | No concerns | No concerns | No concerns | Major concerns | Very low | ["Within-study bias","Incoherence"] |
| AP:EA-MED | 0 | Some concerns | Low risk | No concerns | Major concerns | No concerns | Major concerns | Very low | ["Within-study bias","Imprecision","Incoherence"] |
| AP:EA-Mox | 0 | Some concerns | Low risk | No concerns | No concerns | No concerns | Major concerns | Very low | ["Within-study bias","Incoherence"] |
| AP:EA-TUI | 0 | Some concerns | Low risk | No concerns | No concerns | No concerns | Major concerns | Very low | ["Within-study bias","Incoherence"] |

| Comparison | Number of studies | Within-study bias | Reporting bias | Indirectness | Imprecision | Heterogeneity | Incoherence | Confidence rating | Reason(s) for downgrading |
|---|---|---|---|---|---|---|---|---|---|
| AP:TUI | 0 | Some concerns | Low risk | No concerns | Major concerns | No concerns | Major concerns | Very low | ["Within-study bias","Imprecision","Incoherence"] |
| AP:WNM-APT | 0 | Some concerns | Low risk | No concerns | Some concerns | No concerns | Major concerns | Very low | ["Within-study bias","Imprecision","Incoherence"] |
| AP:WNM-TUI | 0 | Some concerns | Low risk | No concerns | No concerns | No concerns | Major concerns | Very low | ["Within-study bias","Incoherence"] |
| AP-APT:AP-COM | 0 | Some concerns | Low risk | No concerns | Major concerns | No concerns | Major concerns | Very low | ["Within-study bias","Imprecision","Incoherence"] |
| AP-APT:AP-MED | 0 | Some concerns | Low risk | No concerns | Major concerns | No concerns | Major concerns | Very low | ["Within-study bias","Imprecision","Incoherence"] |
| AP-APT:AP-Mox | 0 | Some concerns | Low risk | No concerns | Major concerns | No concerns | Major concerns | Very low | ["Within-study bias","Imprecision","Incoherence"] |
| AP-APT:AP-TUI | 0 | Some concerns | Low risk | No concerns | Major concerns | No concerns | Major concerns | Very low | ["Within-study bias","Imprecision","Incoherence"] |
| AP-APT:AP-TUI-MED | 0 | Some concerns | Low risk | No concerns | Major concerns | No concerns | Major concerns | Very low | ["Within-study bias","Imprecision","Incoherence"] |
| AP-APT:COM | 0 | Some concerns | Low risk | No concerns | No concerns | No concerns | Major concerns | Very low | ["Within-study bias","Incoherence"] |
| AP-APT:EA | 0 | Some concerns | Low risk | No concerns | Major concerns | No concerns | Major concerns | Very low | ["Within-study bias","Imprecision","Incoherence"] |
| AP-APT:EA-APT | 0 | Some concerns | Low risk | No concerns | Major concerns | No concerns | Major concerns | Very low | ["Within-study bias","Imprecision","Incoherence"] |

| Comparison | Number of studies | Within-study bias | Reporting bias | Indirectness | Imprecision | Heterogeneity | Incoherence | Confidence rating | Reason(s) for downgrading |
|---|---|---|---|---|---|---|---|---|---|
| AP-APT:EA-MED | 0 | Some concerns | Low risk | No concerns | No concerns | No concerns | Major concerns | Very low | ["Within-study bias","Incoherence"] |
| AP-APT:EA-Mox | 0 | Some concerns | Low risk | No concerns | Major concerns | No concerns | Major concerns | Very low | ["Within-study bias","Imprecision","Incoherence"] |
| AP-APT:EA-TUI | 0 | Some concerns | Low risk | No concerns | Major concerns | No concerns | Major concerns | Very low | ["Within-study bias","Imprecision","Incoherence"] |
| AP-APT:MED | 0 | Some concerns | Low risk | No concerns | No concerns | No concerns | Major concerns | Very low | ["Within-study bias","Incoherence"] |
| AP-APT:TUI | 0 | Some concerns | Low risk | No concerns | No concerns | No concerns | Major concerns | Very low | ["Within-study bias","Incoherence"] |
| AP-APT:WNM | 0 | Some concerns | Low risk | No concerns | Major concerns | No concerns | Major concerns | Very low | ["Within-study bias","Imprecision","Incoherence"] |
| AP-APT:WNM-APT | 0 | Some concerns | Low risk | No concerns | Major concerns | No concerns | Major concerns | Very low | ["Within-study bias","Imprecision","Incoherence"] |
| AP-APT:WNM-TUI | 0 | Some concerns | Low risk | No concerns | Major concerns | No concerns | Major concerns | Very low | ["Within-study bias","Imprecision","Incoherence"] |
| AP-COM:AP-MED | 0 | Some concerns | Low risk | No concerns | Major concerns | No concerns | Major concerns | Very low | ["Within-study bias","Imprecision","Incoherence"] |
| AP-COM:AP-Mox | 0 | Some concerns | Low risk | No concerns | Major concerns | No concerns | Major concerns | Very low | ["Within-study bias","Imprecision","Incoherence"] |
| AP-COM:AP-TUI | 0 | Some concerns | Low risk | No concerns | Major concerns | No concerns | Major concerns | Very low | ["Within-study bias","Imprecision","Incoherence"] |

| Comparison | Number of studies | Within-study bias | Reporting bias | Indirectness | Imprecision | Heterogeneity | Incoherence | Confidence rating | Reason(s) for downgrading |
|---|---|---|---|---|---|---|---|---|---|
| AP-COM:AP-TUI-MED | 0 | Some concerns | Low risk | No concerns | Major concerns | No concerns | Major concerns | Very low | ["Within-study bias","Imprecision","Incoherence"] |
| AP-COM:EA | 0 | Major concerns | Low risk | No concerns | Major concerns | No concerns | Major concerns | Very low | ["Within-study bias","Imprecision","Incoherence"] |
| AP-COM:EA-APT | 0 | Some concerns | Low risk | No concerns | Major concerns | No concerns | Major concerns | Very low | ["Within-study bias","Imprecision","Incoherence"] |
| AP-COM:EA-MED | 0 | Some concerns | Low risk | No concerns | No concerns | No concerns | Some concerns | Low | ["Within-study bias","Incoherence"] |
| AP-COM:EA-Mox | 0 | Some concerns | Low risk | No concerns | No concerns | Some concerns | Major concerns | Very low | ["Within-study bias","Heterogeneity","Incoherence"] |
| AP-COM:EA-TUI | 0 | Some concerns | Low risk | No concerns | Major concerns | No concerns | Major concerns | Very low | ["Within-study bias","Imprecision","Incoherence"] |
| AP-COM:MED | 0 | Some concerns | Low risk | No concerns | No concerns | No concerns | Major concerns | Very low | ["Within-study bias","Incoherence"] |
| AP-COM:TUI | 0 | Some concerns | Low risk | No concerns | No concerns | No concerns | Some concerns | Low | ["Within-study bias","Incoherence"] |
| AP-COM:WNM | 0 | Some concerns | Low risk | No concerns | Major concerns | No concerns | Major concerns | Very low | ["Within-study bias","Imprecision","Incoherence"] |
| AP-COM:WNM-APT | 0 | Some concerns | Low risk | No concerns | Major concerns | No concerns | Major concerns | Very low | ["Within-study bias","Imprecision","Incoherence"] |
| AP-COM:WNM-TUI | 0 | Some concerns | Low risk | No concerns | Major concerns | No concerns | Major concerns | Very low | ["Within-study bias","Imprecision","Incoherence"] |

| Comparison | Number of studies | Within-study bias | Reporting bias | Indirectness | Imprecision | Heterogeneity | Incoherence | Confidence rating | Reason(s) for downgrading |
|---|---|---|---|---|---|---|---|---|---|
| AP-MED:AP-Mox | 0 | Some concerns | Low risk | No concerns | Major concerns | No concerns | Major concerns | Very low | ["Within-study bias","Imprecision","Incoherence"] |
| AP-MED:AP-TUI | 0 | Some concerns | Low risk | No concerns | Major concerns | No concerns | Major concerns | Very low | ["Within-study bias","Imprecision","Incoherence"] |
| AP-MED:AP-TUI-MED | 0 | Some concerns | Low risk | No concerns | Major concerns | No concerns | Major concerns | Very low | ["Within-study bias","Imprecision","Incoherence"] |
| AP-MED:COM | 0 | Some concerns | Low risk | No concerns | No concerns | No concerns | Major concerns | Very low | ["Within-study bias","Incoherence"] |
| AP-MED:EA | 0 | Some concerns | Low risk | No concerns | Major concerns | No concerns | Major concerns | Very low | ["Within-study bias","Imprecision","Incoherence"] |
| AP-MED:EA-APT | 0 | Some concerns | Low risk | No concerns | Major concerns | No concerns | Major concerns | Very low | ["Within-study bias","Imprecision","Incoherence"] |
| AP-MED:EA-MED | 0 | Some concerns | Low risk | No concerns | No concerns | No concerns | Major concerns | Very low | ["Within-study bias","Incoherence"] |
| AP-MED:EA-Mox | 0 | Some concerns | Low risk | No concerns | Major concerns | No concerns | Major concerns | Very low | ["Within-study bias","Imprecision","Incoherence"] |
| AP-MED:EA-TUI | 0 | Some concerns | Low risk | No concerns | Major concerns | No concerns | Major concerns | Very low | ["Within-study bias","Imprecision","Incoherence"] |
| AP-MED:TUI | 0 | Some concerns | Low risk | No concerns | No concerns | No concerns | Some concerns | Low | ["Within-study bias","Incoherence"] |
| AP-MED:WNM | 0 | Some concerns | Low risk | No concerns | Some concerns | No concerns | Major concerns | Very low | ["Within-study bias","Imprecision","Incoherence"] |

| Comparison | Number of studies | Within-study bias | Reporting bias | Indirectness | Imprecision | Heterogeneity | Incoherence | Confidence rating | Reason(s) for downgrading |
|---|---|---|---|---|---|---|---|---|---|
| AP-MED:WNM-APT | 0 | Some concerns | Low risk | No concerns | Major concerns | No concerns | Major concerns | Very low | ["Within-study bias","Imprecision","Incoherence"] |
| AP-MED:WNM-TUI | 0 | Some concerns | Low risk | No concerns | Major concerns | No concerns | Major concerns | Very low | ["Within-study bias","Imprecision","Incoherence"] |
| AP-Mox:AP-TUI | 0 | Some concerns | Low risk | No concerns | Major concerns | No concerns | Major concerns | Very low | ["Within-study bias","Imprecision","Incoherence"] |
| AP-Mox:AP-TUI-MED | 0 | Some concerns | Low risk | No concerns | Major concerns | No concerns | Major concerns | Very low | ["Within-study bias","Imprecision","Incoherence"] |
| AP-Mox:COM | 0 | Major concerns | Low risk | No concerns | No concerns | No concerns | Major concerns | Very low | ["Within-study bias","Incoherence"] |
| AP-Mox:EA | 0 | Some concerns | Low risk | No concerns | Major concerns | No concerns | Major concerns | Very low | ["Within-study bias","Imprecision","Incoherence"] |
| AP-Mox:EA-APT | 0 | Some concerns | Low risk | No concerns | Major concerns | No concerns | Major concerns | Very low | ["Within-study bias","Imprecision","Incoherence"] |
| AP-Mox:EA-MED | 0 | Some concerns | Low risk | No concerns | No concerns | No concerns | Some concerns | Low | ["Within-study bias","Incoherence"] |
| AP-Mox:EA-Mox | 0 | Some concerns | Low risk | No concerns | Major concerns | No concerns | Major concerns | Very low | ["Within-study bias","Imprecision","Incoherence"] |
| AP-Mox:EA-TUI | 0 | Some concerns | Low risk | No concerns | Major concerns | No concerns | Major concerns | Very low | ["Within-study bias","Imprecision","Incoherence"] |
| AP-Mox:TUI | 0 | Some concerns | Low risk | No concerns | No concerns | No concerns | Some concerns | Low | ["Within-study bias","Incoherence"] |

| Comparison | Number of studies | Within-study bias | Reporting bias | Indirectness | Imprecision | Heterogeneity | Incoherence | Confidence rating | Reason(s) for downgrading |
|---|---|---|---|---|---|---|---|---|---|
| AP-Mox:WNM | 0 | Some concerns | Low risk | No concerns | No concerns | No concerns | Some concerns | Low | ["Within-study bias","Incoherence"] |
| AP-Mox:WNM-APT | 0 | Some concerns | Low risk | No concerns | Major concerns | No concerns | Major concerns | Very low | ["Within-study bias","Imprecision","Incoherence"] |
| AP-Mox:WNM-TUI | 0 | Some concerns | Low risk | No concerns | Major concerns | No concerns | Major concerns | Very low | ["Within-study bias","Imprecision","Incoherence"] |
| AP-TUI:AP-TUI-MED | 0 | Some concerns | Low risk | No concerns | Major concerns | No concerns | Major concerns | Very low | ["Within-study bias","Imprecision","Incoherence"] |
| AP-TUI:COM | 0 | Some concerns | Low risk | No concerns | No concerns | No concerns | Major concerns | Very low | ["Within-study bias","Incoherence"] |
| AP-TUI:EA | 0 | Some concerns | Low risk | No concerns | Major concerns | No concerns | Major concerns | Very low | ["Within-study bias","Imprecision","Incoherence"] |
| AP-TUI:EA-APT | 0 | Some concerns | Low risk | No concerns | Major concerns | No concerns | Major concerns | Very low | ["Within-study bias","Imprecision","Incoherence"] |
| AP-TUI:EA-MED | 0 | Some concerns | Low risk | No concerns | No concerns | No concerns | Some concerns | Low | ["Within-study bias","Incoherence"] |
| AP-TUI:EA-Mox | 0 | Some concerns | Low risk | No concerns | No concerns | No concerns | Some concerns | Low | ["Within-study bias","Incoherence"] |
| AP-TUI:EA-TUI | 0 | Some concerns | Low risk | No concerns | Major concerns | No concerns | Major concerns | Very low | ["Within-study bias","Imprecision","Incoherence"] |
| AP-TUI:WNM | 0 | Some concerns | Low risk | No concerns | Major concerns | No concerns | Major concerns | Very low | ["Within-study bias","Imprecision","Incoherence"] |

| Comparison | Number of studies | Within-study bias | Reporting bias | Indirectness | Imprecision | Heterogeneity | Incoherence | Confidence rating | Reason(s) for downgrading |
|---|---|---|---|---|---|---|---|---|---|
| AP-TUI:WNM-APT | 0 | Some concerns | Low risk | No concerns | Major concerns | No concerns | Major concerns | Very low | ["Within-study bias","Imprecision","Incoherence"] |
| AP-TUI:WNM-TUI | 0 | Some concerns | Low risk | No concerns | Major concerns | No concerns | Major concerns | Very low | ["Within-study bias","Imprecision","Incoherence"] |
| AP-TUI-MED:COM | 0 | Some concerns | Low risk | No concerns | Major concerns | No concerns | Major concerns | Very low | ["Within-study bias","Imprecision","Incoherence"] |
| AP-TUI-MED:EA | 0 | No concerns | Low risk | No concerns | Major concerns | No concerns | Major concerns | Very low | ["Imprecision","Incoherence"] |
| AP-TUI-MED:EA-APT | 0 | Major concerns | Low risk | No concerns | Major concerns | No concerns | Major concerns | Very low | ["Within-study bias","Imprecision","Incoherence"] |
| AP-TUI-MED:EA-MED | 0 | No concerns | Low risk | No concerns | Major concerns | No concerns | Major concerns | Low | ["Incoherence"] |
| AP-TUI-MED:EA-Mox | 0 | Major concerns | Low risk | No concerns | No concerns | No concerns | Major concerns | Very low | ["Within-study bias","Incoherence"] |
| AP-TUI-MED:EA-TUI | 0 | Major concerns | Low risk | No concerns | Major concerns | No concerns | Major concerns | Very low | ["Within-study bias","Imprecision","Incoherence"] |
| AP-TUI-MED:TUI | 0 | Some concerns | Low risk | No concerns | Major concerns | No concerns | Major concerns | Very low | ["Within-study bias","Imprecision","Incoherence"] |
| AP-TUI-MED:WNM | 0 | Some concerns | Low risk | No concerns | Major concerns | No concerns | Major concerns | Very low | ["Within-study bias","Imprecision","Incoherence"] |
| AP-TUI-MED:WNM-APT | 0 | Some concerns | Low risk | No concerns | Major concerns | No concerns | Major concerns | Very low | ["Within-study bias","Imprecision"] |

| Comparison | Number of studies | Within-study bias | Reporting bias | Indirectness | Imprecision | Heterogeneity | Incoherence | Confidence rating | Reason(s) for downgrading |
|---|---|---|---|---|---|---|---|---|---|
| AP-TUI-MED:WNM-TUI | 0 | Some concerns | Low risk | No concerns | Major concerns | No concerns | Major concerns | Very low | ["Within-study bias","Imprecision","Incoherence"] |
| COM:EA | 0 | Major concerns | Low risk | No concerns | No concerns | No concerns | Some concerns | Very low | ["Within-study bias","Incoherence"] |
| COM:EA-APT | 0 | Some concerns | Low risk | No concerns | No concerns | No concerns | Some concerns | Low | ["Within-study bias","Incoherence"] |
| COM:EA-MED | 0 | Some concerns | Low risk | No concerns | Major concerns | No concerns | Some concerns | Very low | ["Within-study bias","Incoherence"] |
| COM:EA-Mox | 0 | Major concerns | Low risk | No concerns | No concerns | No concerns | Major concerns | Very low | ["Within-study bias","Incoherence"] |
| COM:EA-TUI | 0 | Major concerns | Low risk | No concerns | No concerns | No concerns | Major concerns | Very low | ["Within-study bias","Incoherence"] |
| COM:MED | 0 | Some concerns | Low risk | No concerns | Major concerns | No concerns | Major concerns | Very low | ["Within-study bias","Imprecision","Incoherence"] |
| COM:TUI | 0 | Some concerns | Low risk | No concerns | Major concerns | No concerns | Major concerns | Very low | ["Within-study bias","Imprecision","Incoherence"] |
| COM:WNM | 0 | Some concerns | Low risk | No concerns | Major concerns | No concerns | Major concerns | Very low | ["Within-study bias","Imprecision","Incoherence"] |
| COM:WNM-APT | 0 | Some concerns | Low risk | No concerns | No concerns | No concerns | Major concerns | Very low | ["Within-study bias","Incoherence"] |
| COM:WNM-TUI | 0 | Some concerns | Low risk | No concerns | No concerns | No concerns | Major concerns | Very low | ["Within-study bias","Incoherence"] |

| Comparison | Number of studies | Within-study bias | Reporting bias | Indirectness | Imprecision | Heterogeneity | Incoherence | Confidence rating | Reason(s) for downgrading |
|---|---|---|---|---|---|---|---|---|---|
| EA:EA-MED | 0 | Some concerns | Low risk | No concerns | No concerns | No concerns | Major concerns | Very low | ["Within-study bias","Incoherence"] |
| EA:TUI | 0 | Some concerns | Low risk | No concerns | No concerns | No concerns | Some concerns | Low | ["Within-study bias","Incoherence"] |
| EA:WNM | 0 | Some concerns | Low risk | No concerns | Major concerns | No concerns | Major concerns | Very low | ["Within-study bias","Imprecision","Incoherence"] |
| EA:WNM-APT | 0 | Some concerns | Low risk | No concerns | Major concerns | No concerns | Major concerns | Very low | ["Within-study bias","Imprecision","Incoherence"] |
| EA:WNM-TUI | 0 | Some concerns | Low risk | No concerns | Major concerns | No concerns | Major concerns | Very low | ["Within-study bias","Imprecision","Incoherence"] |
| EA-APT:EA-MED | 0 | Some concerns | Low risk | No concerns | No concerns | No concerns | Some concerns | Low | ["Within-study bias","Incoherence"] |
| EA-APT:EA-Mox | 0 | Some concerns | Low risk | No concerns | Major concerns | No concerns | Major concerns | Very low | ["Within-study bias","Imprecision","Incoherence"] |
| EA-APT:EA-TUI | 0 | Some concerns | Low risk | No concerns | Major concerns | No concerns | Major concerns | Very low | ["Within-study bias","Imprecision","Incoherence"] |
| EA-APT:MED | 0 | Some concerns | Low risk | No concerns | No concerns | No concerns | Some concerns | Low | ["Within-study bias","Incoherence"] |
| EA-APT:TUI | 0 | Some concerns | Low risk | No concerns | No concerns | No concerns | Some concerns | Low | ["Within-study bias","Incoherence"] |
| EA-APT:WNM | 0 | Some concerns | Low risk | No concerns | No concerns | No concerns | Some concerns | Low | ["Within-study bias","Incoherence"] |

| Comparison | Number of studies | Within-study bias | Reporting bias | Indirectness | Imprecision | Heterogeneity | Incoherence | Confidence rating | Reason(s) for downgrading |
|---|---|---|---|---|---|---|---|---|---|
| EA-APT:WNM-APT | 0 | Some concerns | Low risk | No concerns | Major concerns | No concerns | Major concerns | Very low | ["Within-study bias","Imprecision","Incoherence"] |
| EA-APT:WNM-TUI | 0 | Some concerns | Low risk | No concerns | Major concerns | No concerns | Major concerns | Very low | ["Within-study bias","Imprecision","Incoherence"] |
| EA-MED:EA-Mox | 0 | Some concerns | Low risk | No concerns | No concerns | No concerns | Major concerns | Very low | ["Within-study bias","Incoherence"] |
| EA-MED:EA-TUI | 0 | Some concerns | Low risk | No concerns | No concerns | No concerns | Major concerns | Very low | ["Within-study bias","Incoherence"] |
| EA-MED:TUI | 0 | Some concerns | Low risk | No concerns | Major concerns | No concerns | Major concerns | Very low | ["Within-study bias","Imprecision","Incoherence"] |
| EA-MED:WNM | 0 | Some concerns | Low risk | No concerns | Major concerns | No concerns | Major concerns | Very low | ["Within-study bias","Imprecision","Incoherence"] |
| EA-MED:WNM-APT | 0 | Some concerns | Low risk | No concerns | No concerns | No concerns | Major concerns | Very low | ["Within-study bias","Incoherence"] |
| EA-MED:WNM-TUI | 0 | Some concerns | Low risk | No concerns | No concerns | No concerns | Some concerns | Low | ["Within-study bias","Incoherence"] |
| EA-Mox:EA-TUI | 0 | Some concerns | Low risk | No concerns | Major concerns | No concerns | Major concerns | Very low | ["Within-study bias","Imprecision","Incoherence"] |
| EA-Mox:MED | 0 | Major concerns | Low risk | No concerns | No concerns | No concerns | Major concerns | Very low | ["Within-study bias","Incoherence"] |
| EA-Mox:TUI | 0 | Some concerns | Low risk | No concerns | No concerns | No concerns | Major concerns | Very low | ["Within-study bias","Incoherence"] |

| Comparison | Number of studies | Within-study bias | Reporting bias | Indirectness | Imprecision | Heterogeneity | Incoherence | Confidence rating | Reason(s) for downgrading |
|---|---|---|---|---|---|---|---|---|---|
| EA-Mox:WNM | 0 | Some concerns | Low risk | No concerns | No concerns | No concerns | Major concerns | Very low | ["Within-study bias","Incoherence"] |
| EA-Mox:WNM-APT | 0 | Some concerns | Low risk | No concerns | Major concerns | No concerns | Major concerns | Very low | ["Within-study bias","Imprecision","Incoherence"] |
| EA-Mox:WNM-TUI | 0 | Some concerns | Low risk | No concerns | Major concerns | No concerns | Major concerns | Very low | ["Within-study bias","Imprecision","Incoherence"] |
| EA-TUI:MED | 0 | Major concerns | Low risk | No concerns | No concerns | No concerns | Major concerns | Very low | ["Within-study bias","Incoherence"] |
| EA-TUI:TUI | 0 | Some concerns | Low risk | No concerns | No concerns | No concerns | Some concerns | Low | ["Within-study bias","Incoherence"] |
| EA-TUI:WNM | 0 | Some concerns | Low risk | No concerns | No concerns | No concerns | Some concerns | Low | ["Within-study bias","Incoherence"] |
| EA-TUI:WNM-APT | 0 | Some concerns | Low risk | No concerns | Major concerns | No concerns | Major concerns | Very low | ["Within-study bias","Imprecision","Incoherence"] |
| EA-TUI:WNM-TUI | 0 | Some concerns | Low risk | No concerns | Major concerns | No concerns | Major concerns | Very low | ["Within-study bias","Imprecision","Incoherence"] |
| MED:TUI | 0 | Some concerns | Low risk | No concerns | Major concerns | No concerns | Major concerns | Very low | ["Within-study bias","Imprecision","Incoherence"] |
| TUI:WNM | 0 | Some concerns | Low risk | No concerns | Major concerns | No concerns | Major concerns | Very low | ["Within-study bias","Imprecision","Incoherence"] |
| TUI:WNM-APT | 0 | Some concerns | Low risk | No concerns | No concerns | No concerns | Some concerns | Low | ["Within-study bias","Incoherence"] |

| Comparison | Number of studies | Within-study bias | Reporting bias | Indirectness | Imprecision | Heterogeneity | Incoherence | Confidence rating | Reason(s) for downgrading |
|---|---|---|---|---|---|---|---|---|---|
| WNM-APT:WNM-TUI | 0 | Some concerns | Low risk | No concerns | Major concerns | No concerns | Major concerns | Very low | ["Within-study bias","Imprecision","Incoherence"] |

Supplementary Table S4. CINeMA-based certainty of evidence (GRADE ratings) for ESCV.

| Comparison | Number of studies | Within-study bias | Reporting bias | Indirectness | Imprecision | Heterogeneity | Incoherence | Confidence rating | Reason(s) for downgrading |
|---|---|---|---|---|---|---|---|---|---|
| AP:AP-APT | 1 | Some concerns | Low risk | No concerns | Major concerns | No concerns | Major concerns | Very low | ["Within-study bias","Imprecision","Incoherence"] |
| AP:AP-COM | 1 | Some concerns | Low risk | No concerns | Major concerns | No concerns | Major concerns | Very low | ["Within-study bias","Imprecision","Incoherence"] |
| AP:AP-MED | 1 | Some concerns | Low risk | No concerns | Major concerns | No concerns | Major concerns | Very low | ["Within-study bias","Imprecision","Incoherence"] |
| AP:AP-Mox | 3 | Some concerns | Low risk | No concerns | No concerns | Major concerns | Major concerns | Very low | ["Within-study bias","Heterogeneity","Incoherence"] |
| AP:AP-TUI | 2 | Some concerns | Low risk | No concerns | Major concerns | No concerns | Major concerns | Very low | ["Within-study bias","Imprecision","Incoherence"] |
| AP-COM:COM | 1 | Some concerns | Low risk | No concerns | No concerns | Major concerns | Major concerns | Very low | ["Within-study bias","Heterogeneity","Incoherence"] |

| Comparison | Number of studies | Within-study bias | Reporting bias | Indirectness | Imprecision | Heterogeneity | Incoherence | Confidence rating | Reason(s) for downgrading |
|---|---|---|---|---|---|---|---|---|---|
| AP-Mox:MED | 1 | Some concerns | Low risk | No concerns | No concerns | Major concerns | Major concerns | Very low | ["Within-study bias","Heterogeneity","Incoherence"] |
| AP-Mox-TUI: TUI | 1 | No concerns | Low risk | No concerns | No concerns | Some concerns | Some concerns | low | ["Heterogeneity","Incoherence"] |
| EA-MED:MED | 2 | Some concerns | Low risk | No concerns | Major concerns | No concerns | Major concerns | Very low | ["Within-study bias","Imprecision","Incoherence"] |
| MED:WNM-APT | 1 | Some concerns | Low risk | No concerns | Major concerns | No concerns | Major concerns | Very low | ["Within-study bias","Imprecision","Incoherence"] |
| TUI:WNM-TUI | 1 | No concerns | Low risk | No concerns | No concerns | Some concerns | Some concerns | low | ["Heterogeneity","Incoherence"] |
| WNM:WNM-APT | 1 | Some concerns | Low risk | No concerns | Major concerns | No concerns | Major concerns | Very low | ["Within-study bias","Imprecision","Incoherence"] |
| WNM:WNM-TUI | 1 | Some concerns | Low risk | No concerns | Major concerns | No concerns | Major concerns | Very low | ["Within-study bias","Imprecision","Incoherence"] |
| AP:AP-Mox-TUI | 0 | Some concerns | Low risk | No concerns | Major concerns | No concerns | Major concerns | Very low | ["Within-study bias","Imprecision","Incoherence"] |
| AP:COM | 0 | Some concerns | Low risk | No concerns | Major concerns | No concerns | Major concerns | Very low | ["Within-study bias","Imprecision","Incoherence"] |
| AP:EA-MED | 0 | Some concerns | Low risk | No concerns | Major concerns | No concerns | Major concerns | Very low | ["Within-study bias","Imprecision","Incoherence"] |
| AP:MED | 0 | Some concerns | Low risk | No concerns | Major concerns | No concerns | Major concerns | Very low | ["Within-study bias","Imprecision","Incoherence"] |

| Comparison | Number of studies | Within-study bias | Reporting bias | Indirectness | Imprecision | Heterogeneity | Incoherence | Confidence rating | Reason(s) for downgrading |
|---|---|---|---|---|---|---|---|---|---|
| AP:TUI | 0 | Some concerns | Low risk | No concerns | Major concerns | No concerns | Major concerns | Very low | ["Within-study bias","Imprecision","Incoherence"] |
| AP:WNM | 0 | Some concerns | Low risk | No concerns | Major concerns | No concerns | Major concerns | Very low | ["Within-study bias","Imprecision","Incoherence"] |
| AP:WNM-APT | 0 | Some concerns | Low risk | No concerns | Major concerns | No concerns | Major concerns | Very low | ["Within-study bias","Imprecision","Incoherence"] |
| AP:WNM-TUI | 0 | Some concerns | Low risk | No concerns | Major concerns | No concerns | Major concerns | Very low | ["Within-study bias","Imprecision","Incoherence"] |
| AP-APT:AP-COM | 0 | Some concerns | Low risk | No concerns | Major concerns | No concerns | Major concerns | Very low | ["Within-study bias","Imprecision","Incoherence"] |
| AP-APT:AP-MED | 0 | Some concerns | Low risk | No concerns | Major concerns | No concerns | Major concerns | Very low | ["Within-study bias","Imprecision","Incoherence"] |
| AP-APT:AP-Mox | 0 | Some concerns | Low risk | No concerns | Major concerns | No concerns | Major concerns | Very low | ["Within-study bias","Imprecision","Incoherence"] |
| AP-APT:AP-Mox-TUI | 0 | Some concerns | Low risk | No concerns | Major concerns | No concerns | Major concerns | Very low | ["Within-study bias","Imprecision","Incoherence"] |
| AP-APT:AP-TUI | 0 | Some concerns | Low risk | No concerns | Major concerns | No concerns | Major concerns | Very low | ["Within-study bias","Imprecision","Incoherence"] |
| AP-APT:COM | 0 | Some concerns | Low risk | No concerns | Major concerns | No concerns | Major concerns | Very low | ["Within-study bias","Imprecision","Incoherence"] |
| AP-APT:EA-MED | 0 | Some concerns | Low risk | No concerns | Major concerns | No concerns | Major concerns | Very low | ["Within-study bias","Imprecision","Incoherence"] |

| Comparison | Number of studies | Within-study bias | Reporting bias | Indirectness | Imprecision | Heterogeneity | Incoherence | Confidence rating | Reason(s) for downgrading |
|---|---|---|---|---|---|---|---|---|---|
| AP-APT:MED | 0 | Some concerns | Low risk | No concerns | Some concerns | Some concerns | Major concerns | Very low | ["Within-study bias","Imprecision","Heterogeneity","Incoherence"] |
| AP-APT:TUI | 0 | Some concerns | Low risk | No concerns | Major concerns | No concerns | Major concerns | Very low | ["Within-study bias","Imprecision","Incoherence"] |
| AP-APT:WNM | 0 | Some concerns | Low risk | No concerns | Major concerns | No concerns | Major concerns | Very low | ["Within-study bias","Imprecision","Incoherence"] |
| AP-APT:WNM-APT | 0 | Some concerns | Low risk | No concerns | Major concerns | No concerns | Major concerns | Very low | ["Within-study bias","Imprecision","Incoherence"] |
| AP-APT:WNM-TUI | 0 | Some concerns | Low risk | No concerns | Major concerns | No concerns | Major concerns | Very low | ["Within-study bias","Imprecision","Incoherence"] |
| AP-COM:AP-MED | 0 | Some concerns | Low risk | No concerns | Major concerns | No concerns | Major concerns | Very low | ["Within-study bias","Imprecision","Incoherence"] |
| AP-COM:AP-Mox | 0 | Some concerns | Low risk | No concerns | Major concerns | No concerns | Major concerns | Very low | ["Within-study bias","Imprecision","Incoherence"] |
| AP-COM:AP-Mox-TUI | 0 | Some concerns | Low risk | No concerns | Major concerns | No concerns | Major concerns | Very low | ["Within-study bias","Imprecision","Incoherence"] |
| AP-COM:AP-TUI | 0 | Some concerns | Low risk | No concerns | Major concerns | No concerns | Major concerns | Very low | ["Within-study bias","Imprecision","Incoherence"] |
| AP-COM:EA-MED | 0 | Some concerns | Low risk | No concerns | Major concerns | No concerns | Major concerns | Very low | ["Within-study bias","Imprecision","Incoherence"] |
| AP-COM:MED | 0 | Some concerns | Low risk | No concerns | No concerns | Major concerns | Major concerns | Very low | ["Within-study bias","Heterogeneity","Incoherence"] |

| Comparison | Number of studies | Within-study bias | Reporting bias | Indirectness | Imprecision | Heterogeneity | Incoherence | Confidence rating | Reason(s) for downgrading |
|---|---|---|---|---|---|---|---|---|---|
| AP-COM:TUI | 0 | Some concerns | Low risk | No concerns | Major concerns | No concerns | Major concerns | Very low | ["Within-study bias","Imprecision","Incoherence"] |
| AP-COM:WNM | 0 | Some concerns | Low risk | No concerns | Major concerns | No concerns | Major concerns | Very low | ["Within-study bias","Imprecision","Incoherence"] |
| AP-COM:WNM-APT | 0 | Some concerns | Low risk | No concerns | Major concerns | No concerns | Major concerns | Very low | ["Within-study bias","Imprecision","Incoherence"] |
| AP-COM:WNM-TUI | 0 | Some concerns | Low risk | No concerns | Major concerns | No concerns | Major concerns | Very low | ["Within-study bias","Imprecision","Incoherence"] |
| AP-MED:AP-Mox | 0 | Some concerns | Low risk | No concerns | Major concerns | No concerns | Major concerns | Very low | ["Within-study bias","Imprecision","Incoherence"] |
| AP-MED:AP-Mox-TUI | 0 | Some concerns | Low risk | No concerns | Major concerns | No concerns | Major concerns | Very low | ["Within-study bias","Imprecision","Incoherence"] |
| AP-MED:AP-TUI | 0 | Some concerns | Low risk | No concerns | Major concerns | No concerns | Major concerns | Very low | ["Within-study bias","Imprecision","Incoherence"] |
| AP-MED:COM | 0 | Some concerns | Low risk | No concerns | Major concerns | No concerns | Major concerns | Very low | ["Within-study bias","Imprecision","Incoherence"] |
| AP-MED:EA-MED | 0 | Some concerns | Low risk | No concerns | Major concerns | No concerns | Major concerns | Very low | ["Within-study bias","Imprecision","Incoherence"] |
| AP-MED:MED | 0 | Some concerns | Low risk | No concerns | Some concerns | Some concerns | Major concerns | Very low | ["Within-study bias","Imprecision","Heterogeneity","Incoherence"] |
| AP-MED:TUI | 0 | Some concerns | Low risk | No concerns | Major concerns | No concerns | Major concerns | Very low | ["Within-study bias","Imprecision","Incoherence"] |

| Comparison | Number of studies | Within-study bias | Reporting bias | Indirectness | Imprecision | Heterogeneity | Incoherence | Confidence rating | Reason(s) for downgrading |
|---|---|---|---|---|---|---|---|---|---|
| AP-MED:WNM | 0 | Some concerns | Low risk | No concerns | Major concerns | No concerns | Major concerns | Very low | ["Within-study bias","Imprecision","Incoherence"] |
| AP-MED:WNM-APT | 0 | Some concerns | Low risk | No concerns | Major concerns | No concerns | Major concerns | Very low | ["Within-study bias","Imprecision","Incoherence"] |
| AP-MED:WNM-TUI | 0 | Some concerns | Low risk | No concerns | Major concerns | No concerns | Major concerns | Very low | ["Within-study bias","Imprecision","Incoherence"] |
| AP-Mox:AP-Mox-TUI | 0 | Some concerns | Low risk | No concerns | Major concerns | No concerns | Major concerns | Very low | ["Within-study bias","Imprecision","Incoherence"] |
| AP-Mox:AP-TUI | 0 | Some concerns | Low risk | No concerns | Major concerns | No concerns | Major concerns | Very low | ["Within-study bias","Imprecision","Incoherence"] |
| AP-Mox:COM | 0 | Some concerns | Low risk | No concerns | No concerns | Major concerns | Major concerns | Very low | ["Within-study bias","Heterogeneity","Incoherence"] |
| AP-Mox:EA-MED | 0 | Some concerns | Low risk | No concerns | No concerns | Major concerns | Major concerns | Very low | ["Within-study bias","Heterogeneity","Incoherence"] |
| AP-Mox:TUI | 0 | Some concerns | Low risk | No concerns | Some concerns | Some concerns | Major concerns | Very low | ["Within-study bias","Imprecision","Heterogeneity","Incoherence"] |
| AP-Mox:WNM | 0 | Some concerns | Low risk | No concerns | Some concerns | Some concerns | Major concerns | Very low | ["Within-study bias","Imprecision","Heterogeneity","Incoherence"] |
| AP-Mox:WNM-APT | 0 | Some concerns | Low risk | No concerns | Major concerns | No concerns | Major concerns | Very low | ["Within-study bias","Imprecision","Incoherence"] |
| AP-Mox:WN | 0 | Some | Low risk | No | Major concerns | No | Major concerns | Very low | ["Within-study |

| Comparison | Number of studies | Within-study bias | Reporting bias | Indirectness | Imprecision | Heterogeneity | Incoherence | Confidence rating | Reason(s) for downgrading |
|---|---|---|---|---|---|---|---|---|---|
| M-TUI | | concerns | | concerns | | concerns | | | bias","Imprecision","Incoherence"] |
| AP-Mox-TUI: AP-TUI | 0 | Some concerns | Low risk | No concerns | Major concerns | No concerns | Major concerns | Very low | ["Within-study bias","Imprecision","Incoherence"] |
| AP-Mox-TUI: COM | 0 | Some concerns | Low risk | No concerns | Major concerns | No concerns | Major concerns | Very low | ["Within-study bias","Imprecision","Incoherence"] |
| AP-Mox-TUI: EA-MED | 0 | Some concerns | Low risk | No concerns | Major concerns | No concerns | Major concerns | Very low | ["Within-study bias","Imprecision","Incoherence"] |
| AP-Mox-TUI: MED | 0 | Some concerns | Low risk | No concerns | Major concerns | No concerns | Major concerns | Very low | ["Within-study bias","Imprecision","Incoherence"] |
| AP-Mox-TUI: WNM | 0 | No concerns | Low risk | No concerns | No concerns | No concerns | Major concerns | low | ["Incoherence"] |
| AP-Mox-TUI: WNM-APT | 0 | No concerns | Low risk | No concerns | Some concerns | No concerns | Some concerns | low | ["Imprecision","Incoherence"] |
| AP-Mox-TUI: WNM-TUI | 0 | No concerns | Low risk | No concerns | Some concerns | No concerns | Some concerns | low | ["Imprecision","Incoherence"] |
| AP-TUI:COM | 0 | Some concerns | Low risk | No concerns | Major concerns | No concerns | Major concerns | Very low | ["Within-study bias","Imprecision","Incoherence"] |
| AP-TUI:EA-MED | 0 | Some concerns | Low risk | No concerns | Major concerns | No concerns | Major concerns | Very low | ["Within-study bias","Imprecision","Incoherence"] |
| AP-TUI:MED | 0 | Some concerns | Low risk | No concerns | Some concerns | Some concerns | Major concerns | Very low | ["Within-study bias","Imprecision","Heterogeneity","Incoherence"] |

| Comparison | Number of studies | Within-study bias | Reporting bias | Indirectness | Imprecision | Heterogeneity | Incoherence | Confidence rating | Reason(s) for downgrading |
|---|---|---|---|---|---|---|---|---|---|
| AP-TUI:TUI | 0 | Some concerns | Low risk | No concerns | Major concerns | No concerns | Major concerns | Very low | ["Within-study bias","Imprecision","Incoherence"] |
| AP-TUI:WNM | 0 | Some concerns | Low risk | No concerns | Major concerns | No concerns | Major concerns | Very low | ["Within-study bias","Imprecision","Incoherence"] |
| AP-TUI:WNM-APT | 0 | Some concerns | Low risk | No concerns | Major concerns | No concerns | Major concerns | Very low | ["Within-study bias","Imprecision","Incoherence"] |
| AP-TUI:WNM-TUI | 0 | Some concerns | Low risk | No concerns | Major concerns | No concerns | Major concerns | Very low | ["Within-study bias","Imprecision","Incoherence"] |
| COM:EA-MED | 0 | Some concerns | Low risk | No concerns | Major concerns | No concerns | Major concerns | Very low | ["Within-study bias","Imprecision","Incoherence"] |
| COM:MED | 0 | Some concerns | Low risk | No concerns | Major concerns | No concerns | Major concerns | Very low | ["Within-study bias","Imprecision","Incoherence"] |
| COM:TUI | 0 | Some concerns | Low risk | No concerns | Major concerns | No concerns | Major concerns | Very low | ["Within-study bias","Imprecision","Incoherence"] |
| COM:WNM | 0 | Some concerns | Low risk | No concerns | Major concerns | No concerns | Major concerns | Very low | ["Within-study bias","Imprecision","Incoherence"] |
| COM:WNM-APT | 0 | Some concerns | Low risk | No concerns | Major concerns | No concerns | Major concerns | Very low | ["Within-study bias","Imprecision","Incoherence"] |
| COM:WNM-TUI | 0 | Some concerns | Low risk | No concerns | Major concerns | No concerns | Major concerns | Very low | ["Within-study bias","Imprecision","Incoherence"] |
| EA-MED:TUI | 0 | Some concerns | Low risk | No concerns | Major concerns | No concerns | Major concerns | Very low | ["Within-study bias","Imprecision","Incoherence"] |

| Comparison | Number of studies | Within-study bias | Reporting bias | Indirectness | Imprecision | Heterogeneity | Incoherence | Confidence rating | Reason(s) for downgrading |
|---|---|---|---|---|---|---|---|---|---|
| EA-MED:WNM | 0 | Some concerns | Low risk | No concerns | Major concerns | No concerns | Major concerns | Very low | ["Within-study bias","Imprecision","Incoherence"] |
| EA-MED:WNM-APT | 0 | Some concerns | Low risk | No concerns | Major concerns | No concerns | Major concerns | Very low | ["Within-study bias","Imprecision","Incoherence"] |
| EA-MED:WNM-TUI | 0 | Some concerns | Low risk | No concerns | Major concerns | No concerns | Major concerns | Very low | ["Within-study bias","Imprecision","Incoherence"] |
| MED:TUI | 0 | Some concerns | Low risk | No concerns | Major concerns | No concerns | Major concerns | Very low | ["Within-study bias","Imprecision","Incoherence"] |
| MED:WNM | 0 | Some concerns | Low risk | No concerns | Major concerns | No concerns | Major concerns | Very low | ["Within-study bias","Imprecision","Incoherence"] |
| MED:WNM-TUI | 0 | Some concerns | Low risk | No concerns | Major concerns | No concerns | Major concerns | Very low | ["Within-study bias","Imprecision","Incoherence"] |
| TUI:WNM | 0 | No concerns | Low risk | No concerns | Some concerns | No concerns | Some concerns | low | ["Imprecision","Incoherence"] |
| TUI:WNM-APT | 0 | Some concerns | Low risk | No concerns | Major concerns | No concerns | Major concerns | Very low | ["Within-study bias","Imprecision","Incoherence"] |
| WNM-APT:WNM-TUI | 0 | Some concerns | Low risk | No concerns | Major concerns | No concerns | Major concerns | Very low | ["Within-study bias","Imprecision","Incoherence"] |

Supplementary material Figure 1

| Rank 1st | | | | | | | | | | | | | | | | |
|---|---|---|---|---|---|---|---|---|---|---|---|---|---|---|---|---|
| AP-COM | Rank 2nd | | | | | | | | | | | | | | | |
| 0.97 (0.80,1.19) | AP-APT | Rank 3rd | | | | | | | | | | | | | | |
| 1.04 (0.90,1.21) | 1.02 (0.86,1.20) | AP-Mox | Rank 4th | | | | | | | | | | | | | |
| 1.05 (0.84,1.32) | 1.02 (0.80,1.30) | 1.01 (0.83,1.23) | EA-TUI | Rank 5th | | | | | | | | | | | | |
| 1.05 (0.85,1.31) | 1.03 (0.81,1.30) | 1.01 (0.84,1.22) | 1.00 (0.89,1.11) | EA-Mox | Rank 6th | | | | | | | | | | | |
| 1.06 (0.82,1.36) | 1.03 (0.79,1.35) | 1.01 (0.80,1.27) | 0.99 (0.84,1.18) | 1.00 (0.85,1.17) | EA-APT | Rank 7th | | | | | | | | | | |
| 1.08 (0.92,1.28) | 1.05 (0.87,1.28) | 1.04 (0.92,1.18) | 1.03 (0.84,1.26) | 1.03 (0.85,1.24) | 1.03 (0.81,1.30) | WNM-TUI | Rank 8th | | | | | | | | | |
| 1.11 (0.95,1.30) | 1.08 (0.90,1.30) | 1.06 (0.95,1.20) | 0.95 (0.77,1.16) | 0.95 (0.79,1.15) | 0.95 (0.75,1.20) | 0.98 (0.86,1.11) | AP-TUI | Rank 9th | | | | | | | | |
| 1.12 (0.98,1.29) | 1.09 (0.93,1.29) | 0.93 (0.85,1.01) | 0.93 (0.77,1.13) | 0.94 (0.79,1.12) | 0.94 (0.75,1.18) | 0.96 (0.86,1.08) | 0.99 (0.89,1.10) | AP-MED | Rank 10th | | | | | | | |
| 1.17 (0.98,1.39) | 1.14 (0.93,1.38) | 1.12 (0.97,1.29) | 1.11 (0.90,1.37) | 1.11 (0.91,1.35) | 1.10 (0.87,1.40) | 0.93 (0.81,1.07) | 1.05 (0.91,1.21) | 1.04 (0.91,1.18) | WNM-APT | Rank 11th | | | | | | |
| 1.16 (0.94,1.43) | 1.13 (0.90,1.42) | 1.11 (0.93,1.33) | **0.90 (0.83,0.99)** | **0.91 (0.85,0.97)** | 0.91 (0.78,1.05) | 0.93 (0.78,1.12) | 1.05 (0.87,1.25) | 1.03 (0.88,1.22) | 1.00 (0.83,1.21) | EA | Rank 12th | | | | | |
| 1.18 (0.98,1.42) | 1.15 (0.93,1.41) | 1.13 (0.97,1.31) | 0.89 (0.72,1.10) | 0.90 (0.73,1.10) | 0.90 (0.70,1.14) | 0.92 (0.79,1.08) | 1.06 (0.91,1.24) | 1.05 (0.91,1.20) | 0.99 (0.84,1.17) | 0.99 (0.81,1.20) | AP-TUI-MED | Rank 13th | | | | |
| **1.21 (1.00,1.47)** | 1.18 (0.95,1.46) | 1.16 (0.99,1.36) | 0.87 (0.69,1.08) | 0.87 (0.71,1.07) | 1.15 (0.89,1.47) | 0.89 (0.76,1.05) | 1.09 (0.93,1.29) | 1.08 (0.93,1.25) | 0.96 (0.81,1.14) | 1.04 (0.85,1.28) | 1.03 (0.86,1.23) | EA-MED | Rank 14th | | | |
| **0.82 (0.72,0.93)** | **0.84 (0.72,0.98)** | **0.85 (0.80,0.91)** | 0.86 (0.71,1.04) | 0.86 (0.73,1.03) | 0.86 (0.69,1.08) | 0.89 (0.80,0.99) | **0.91 (0.83,1.00)** | **0.92 (0.87,0.97)** | 0.95 (0.84,1.08) | 0.95 (0.81,1.12) | 0.96 (0.84,1.10) | 0.99 (0.86,1.15) | AP | Rank 15th | | |
| **1.26 (1.07,1.48)** | **1.23 (1.02,1.48)** | **1.21 (1.07,1.37)** | 1.20 (0.98,1.47) | 1.19 (0.99,1.44) | 1.19 (0.95,1.50) | **0.86 (0.79,0.93)** | **1.13 (1.00,1.29)** | **1.12 (1.00,1.25)** | 0.93 (0.81,1.06) | 1.08 (0.91,1.30) | 1.07 (0.92,1.25) | 1.04 (0.88,1.22) | 1.03 (0.93,1.14) | WNM | Rank 16th | |
| **1.35 (1.17,1.55)** | **1.31 (1.11,1.55)** | **1.29 (1.18,1.41)** | **1.28 (1.08,1.53)** | **1.28 (1.09,1.51)** | **1.28 (1.03,1.58)** | **0.80 (0.73,0.89)** | **1.21 (1.10,1.34)** | **1.20 (1.12,1.29)** | **0.86 (0.78,0.96)** | **1.16 (1.00,1.35)** | **1.15 (1.02,1.29)** | 1.11 (0.98,1.27) | **1.10 (1.04,1.17)** | 0.93 (0.85,1.03) | MED | Rank 17th |
| **1.44 (1.18,1.76)** | **1.40 (1.12,1.75)** | **1.38 (1.16,1.64)** | **1.37 (1.09,1.73)** | **1.37 (1.09,1.71)** | **1.36 (1.05,1.77)** | **0.75 (0.65,0.87)** | **1.30 (1.11,1.51)** | **1.28 (1.09,1.51)** | **0.81 (0.67,0.97)** | **1.24 (1.00,1.54)** | **1.22 (1.01,1.49)** | 1.19 (0.97,1.45) | **1.18 (1.01,1.38)** | 0.87 (0.75,1.02) | 1.07 (0.92,1.25) | TUI |

Figure S1. League table of the sensitivity analysis for total effectiveness rate after excluding high-risk studies, with comparisons that became non-significant highlighted in blue.
